# Interactions of Autophagy and the Immune System in Health and Diseases

**DOI:** 10.1080/27694127.2022.2119743

**Published:** 2022-10-05

**Authors:** Aarti Pant, Xiaomin Yao, Aude Lavedrine, Christophe Viret, Jacob Dockterman, Swati Chauhan, Chong-Shan Shi, Ravi Manjithaya, Ken Cadwell, Thomas A. Kufer, John H. Kehrl, Jörn Coers, L. David Sibley, Mathias Faure, Gregory A. Taylor, Santosh Chauhan

**Affiliations:** a Autophagy Laboratory, Molecular Biology and Genetics Unit, Jawaharlal Nehru Centre for Advanced Scientific Research, Bengaluru, India; b Kimmel Center for Biology and Medicine at the Skirball Institute, New York University Grossman School of Medicine, New York, New York, USA; c Department of Microbiology, New York University Grossman School of Medicine, New York, New York, USA; d CIRI, Centre International de Recherche en Infectiologie, Université de Lyon, Inserm U1111, Université Claude Bernard Lyon 1, CNRS, UMR5308, ENS de Lyon, F-69007, Lyon, France; e Equipe Labellisée par la Fondation pour la Recherche Médicale, FRM; f Department of Immunology, Duke University, Medical Center, Durham, North Carolina, USA; g Cell biology and Infectious diseases, Institute of Life Sciences, Bhubaneswar, India; h Laboratory of Immunoregulation, Division of Intramural Research, National Institute of Allergy and Infectious Diseases, National Institutes of Health, Bethesda, MD, US; i Neuroscience Unit, Jawaharlal Nehru Centre for Advanced Scientific Research, Bengaluru, India; j Division of Gastroenterology and Hepatology, Department of Medicine, New York University Grossman School of Medicine, New York, New York, USA; k Department of Immunology, Institute of Nutritional Medicine, University of Hohenheim, Stuttgart, Germany; l Department of Molecular Genetics and Microbiology, Duke University, Medical Center, Durham, North Carolina, USA; m Department of Molecular Microbiology, Washington University Sch. Med., St Louis, MO, 63110, USA; n Geriatric Research, Education, and Clinical Center, VA Health Care Center, Durham, North Carolina, USA; o Departments of Medicine, Division of Geriatrics, and Center for the Study of Aging and Human Development, Duke University, Medical Center, Durham, North Carolina, USA; p CSIR–Centre For Cellular And Molecular Biology (CCMB), Hyderabad, Telangana

## Abstract

Autophagy is a highly conserved process that utilizes lysosomes to selectively degrade a variety of intracellular cargo, thus providing quality control over cellular components and maintaining cellular regulatory functions. Autophagy is triggered by multiple stimuli ranging from nutrient starvation to microbial infection. Autophagy extensively shapes and modulates the inflammatory response, the concerted action of immune cells, and secreted mediators aimed to eradicate a microbial infection or to heal sterile tissue damage. Here, we first review how autophagy affects innate immune signaling, cell-autonomous immune defense, and adaptive immunity. Then, we discuss the role of non-canonical autophagy in context of microbial infections and inflammation. Finally, we review how crosstalk between autophagy and inflammation influences infectious diseases as well as metabolic and autoimmune disorders.

## Introduction

### Autophagy: A process that maintains intracellular homeostasis

Macroautophagy (henceforth referred to as autophagy) is a highly conserved self-degradative process that regulates several vital processes in the cell. Autophagy starts with the formation of the autophagosome, a double membrane-bound structure that sequesters cytoplasmic material and delivers it to lysosomes for degradation^1,2^. The molecular details of this process have been comprehensively reviewed elsewhere^3^. Once the cellular contents are degraded, energy is released that can buoy cell survival at critical times of nutrient stress. However, autophagy also plays several key housekeeping roles by clearing damaged organelles, aggregated proteins, and pathogens from cells^1,2,4^. Importantly, autophagy plays a significant role in the regulation of multiple innate and adaptive immune responses, as we review here.

### Inflammation: the double-edged sword

Inflammation is the body’s immune response to harmful stimuli, such as pathogens, toxic substances, or physical/physiological stress. Many regulatory pathways are integrated to help mount a balanced and calculated inflammatory response against each stimulus^5^. Once the stimuli are suppressed, in most cases the inflammatory response is resolved by a set of regulatory feedback mechanisms. However, genetic defects in mechanisms of resolution can cause acute inflammation to become chronic. This results in tissue and organ damage and can eventually lead to chronic inflammatory disorders^5^.

The tissue damage and microbial infection are sensed by pattern-recognition receptors (PRRs) that activate cascades of events resulting in cytokine responses, which are important for the recruitment of adaptive immune cells to the site of injury or infection^6^. The integrated and coordinated program of innate and adaptive immune response determines the fate of the threat^6^. The four major PRR families include Toll-like receptors (TLRs), NOD-like receptors (NLRs), Retinoic acid-inducible gene (RIG)-I-like receptors (RLRs), and C-type lectin receptors (CLRs). These PRRs sense many different types of ligands and initiate several distinct programs of inflammatory cytokine responses.

Recent evidence suggests that autophagy is a key system induced by inflammatory stimuli to modulate inflammatory responses. Indirectly, autophagy suppresses inflammation by targeting the source of inflammation such as microbes and directly degrades innate immune signaling proteins (inflammophagy), keeping inflammation in check^7^. Here, we have focused on the crosstalk that exists between autophagy and immune signaling pathways.

## 1. Autophagy, Innate immune sensors, and cell death

### Crosstalk: TLRs and autophagy

**Figure 1.**
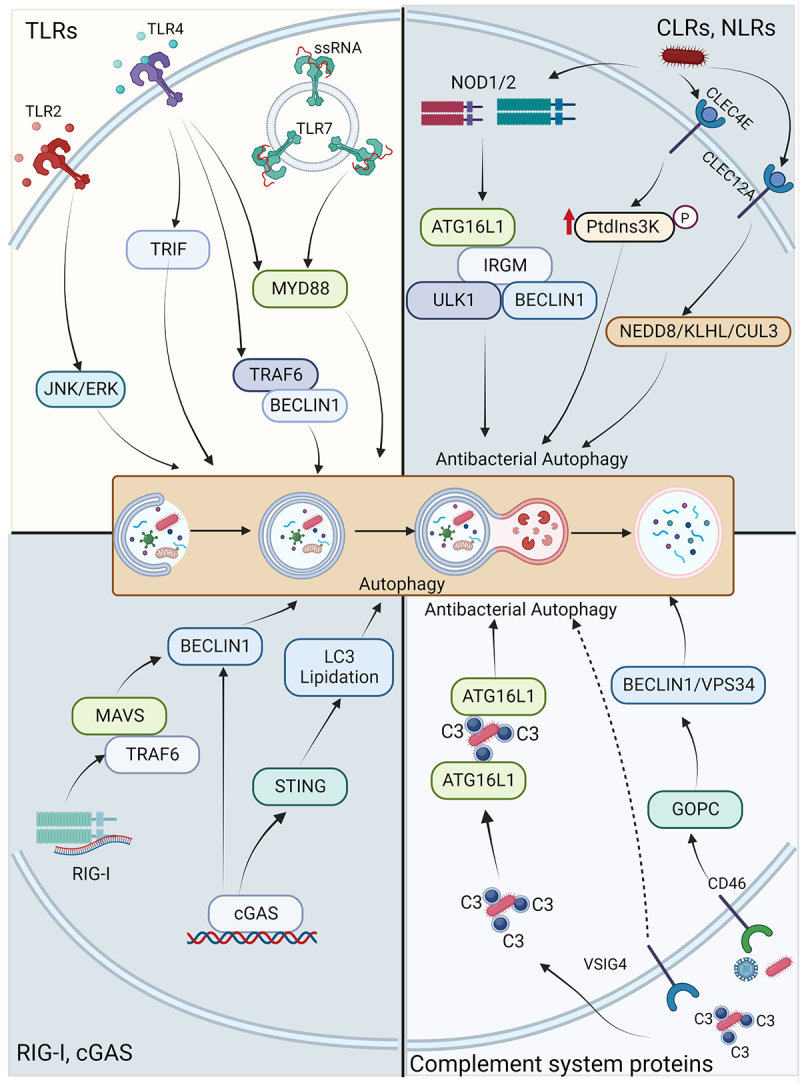
Innate immune signaling pathways activate autophagy. Upon stimulation with PAMPs, DAMPs, and microbes, several PRRs of the different family including TLRs, CLRs, NLRs, RLRs, and cGAS induces autophagy for anti-microbial and homeostatic functions. The pathways are described in text in detail. Illustrations are made using biorender software.

**Figure 2.**
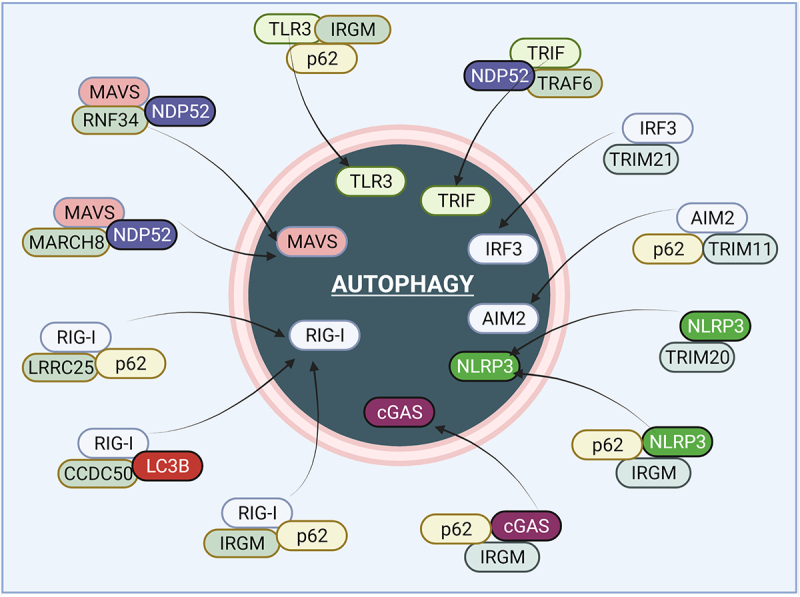
Autophagy degrades several innate immune sensors and intermediary proteins to suppress inflammation. Upon stimulations, autophagy utilizes different molecular mechanisms to degrade PRR pathways sensors and other vital proteins. Autophagy proteins such as p62/SQSTM1, NDP52, IRGM, and TRIM20/21 play a vital role in delivering these inflammatory proteins to autophagosomes for degradation. Several E3 ligases such as RNF34 and MARCH8 play a vital role in the ubiquitination of inflammatory proteins targeted for degradation. The mechanisms are detailed in the text. The illustrations are made using Biorender software.

TLRs are germline-encoded receptors that can sense pathogens through the detection of pathogen-associated molecular patterns (PAMPs) that are conserved among large classes of microbes. TLRs localize in the plasma membrane or within endocytic vesicles to allow for the detection of a broad range of lipids, proteins, lipoproteins, and nucleic acids of microbial origin. TLR-associated signaling relies on the recruitment of the adaptor protein myeloid differentiation primary response 88 (MyD88) and/or in some cases, Toll/interleukin-1 receptor domain-containing adaptor inducing interferon-β (TRIF). Downstream events triggered by TLR signaling pathways include activation of the NF-κB, p38 MAPK, JNK, ERK, and IRF pathways culminating in the production of proinflammatory cytokines and type I interferons. Microbes detection by TLR enhances autophagy that greatly contributes to the associated cellular response to infection^8^ (Figure 1). TLR7 induces LC3 lipidation upon exposure to synthetic single-stranded RNA or during infection with either vesicular stomatitis virus (VSV) or human immunodeficiency virus (HIV)^9,10^. TLR2/1 heterodimer formation enhances autophagy through modulation of the vitamin D receptor signaling during macrophage infection by mycobacteria^11^. The signaling associated with bacterial sensing by TLR2 can activate autophagy in macrophages by activating the ERK and JNK pathways^12–14^ (Figure 1). TLR4 engagement by LPS activates autophagy by mobilizing signaling pathways associated with both MyD88 and TRIF adaptors ^15,16^ (Figure 1). TLR4-TRIF mobilization during *Salmonella* infection can induce TRAF3-dependent activation of TANK-binding kinase (TBK)1, leading to optimized engagement of optineurin in antibacterial autophagy^17^. In macrophages, TLR4-induced autophagy involves the modulation of BECLIN1 via ubiquitination^18^ (Figure 1). Autophagy activation through BECLIN1 regulation also occurs in the presence of TLR2/4 agonists that induce plasminogen activator inhibitor-2 ^19^. Such regulation often attenuates the BECLIN1-BCL2 interaction that negatively regulates autophagy at a steady state. TLR-signaling induced upon mycobacterial infection can also promote autophagosome formation and maturation through induction of the autophagy modulator DRAM1^20^ indicating that TLR engagement can modulate the autophagy flux at distinct steps. At the moment, the mechanisms of autophagy enhancement associated with TLR engagement are incompletely understood and deserve further analysis. What is clear is that multiple TLRs can activate autophagy in various cell types and that the response can be cell-type specific. For instance, unlike in macrophages, TLR7 engagement induces very poor autophagy activation in plasmacytoid dendritic cells (pDC). Because prolonged inflammatory responses can be detrimental to the host, mechanisms exist to dampen such responses when pathogens are being cleared and autophagy contributes to this resolution. Autophagy can thus target components of TLR-associated signaling pathways for degradation^21–24^. For example, depending on the context, the TRIF adaptor can be targeted by the autophagy receptors NDP52, TAX1BP1, or p62/SQSTM1 for lysosomal degradation subsequently to TLR3 and/or TLR4 engagement (Figure 2). Such regulatory selective autophagy can involve the participation of factors as varied as TRAF6 [tumor necrosis factor (TNF) receptor-associated factor 6], IRGM [Immunity related GTPase clade M], TRIM32, or ATG16L1 for its completion^21–23,25^ (Figure 2). Thus, TLR signaling and autophagy cross-regulate to finetune inflammatory and cell-autonomous immune responses to invaders.

### C-Type Lectin Receptors (CLRs) and autophagy

CLRs are pattern recognition receptors expressed predominantly by myeloid cells that participate in immune responses through the sensing of pathogen-associated ligands. Some studies indicate that CLRs can effectively modulate anti-microbial autophagy both positively and negatively. For instance, during *Salmonella* infection of epithelial and immune cells, CLEC12A can selectively promote autophagy by modulating the activity of the NEDD8-KLHL13-CUL3-KLHL9 E3 ligase complex^26^ (Figure 1). CLEC4E can favor antibacterial autophagy as well. In macrophages exposed to *Mycobacterium tuberculosis*, CLEC4E engagement can synergize with TLR4-associated signaling to support antibacterial autophagy by promoting PtdIns3K phosphorylation^27^ (Figure 1), whereas CLEC4E in neutrophils is required for the autophagic activity needed to assist the formation of extracellular traps in response to *Klebsiella pneumoniae*^28^. On the other hand, CLRs can also negatively influence anti-microbial autophagy. In macrophages, Dectin-1, a receptor for β-glucan, can down-regulate autophagy in an NF-κB-dependent manner^29^. Engagement of DC-SIGN by Kaposi’s sarcoma-associated herpes virus (KSHV) blocks autophagy in dendritic cells due to the stabilized activation of signal transducer and activator of transcription 3 (STAT3)^30^. DC-SIGN engagement also negatively regulates the autophagic response of dendritic cells exposed to *Porphyromonas gingivalis*, which is promoted by TLR2-induced signaling^31^.

### Crosstalk between NLRs and autophagy

Many essential intracellular PRRs belong to the family of NLR proteins, which are classified as proteins with a tripartite domain organization with a hallmark NACHT domain that is centrally localized and aids oligomerization. The interaction between NLRs and autophagy is complex and ambivalent, as the activation of certain NLRs by pathogens can induce selective autophagy of invading microbes. However, autophagy also acts as a control mechanism for both NLR activation and the outcome of associated signaling.

### C.1. NLR-induced autophagy for debugging

NOD1 and NOD2 were the first NLR-proteins to be identified as intracellular PRRs that activate NF-κB and MAPK pathways by interacting with the kinase RIPK2. They are sensors for bacterial peptidoglycan^32^ but more recently have also been shown to respond to cell stress^33,34^, manipulation of F-actin dynamics^35,36^, and viruses^37–39^. The first link between NLRs and autophagy was the observation of xenophagy of bacterial pathogens following activation of NOD1 and NOD2. The NOD2 ligand muramyl dipeptide (MDP) can induce autophagy by ATG16L1 activation in epithelial and dendritic cells leading to restriction of *Salmonella* growth^40^, ^41^ (Figure 1). Of note, polymorphisms in both *NOD2* and *ATG16L1* are associated with Crohn’s disease, a severe inflammatory bowel disease. One possible etiologic mechanism is a reduced anti-bacterial function of NOD2. Crohn’s disease-associated mutations in NOD2 and ATG16L1 render these proteins inactive for autophagy induction^42^. Functionally, NOD1 and NOD2 bind ATG16L1^41^ and recruit another Crohn’s disease risk factor IRGM that can act as a scaffold to induce autophagosome formation by recruiting core autophagy proteins^42^ (Figure 1). In dendritic cells, this NOD2/ATG16L1 dependent autophagy also contributes to MHC class II antigen presentation^43^. Induction of autophagy downstream of NOD1/2 is dependent on RIPK2 and its tyrosine kinase activity^40^, albeit recruitment of ATG16L1 to membranes by NOD1/2 was reported to be RIPK2 independent in some cell lines^41^. Besides this effector function that enhances the anti-bacterial response induced by NOD1/2, autophagy can also affect the NOD1/2-induced proinflammatory response, although the molecular details of this pathway are not well understood. For example, inhibition of ATG16L1 in cell lines can reduce NOD2-induced NF-κB activation^44^ but ATG16L1 was also reported to suppress NOD1/2-mediated inflammatory cytokine release by interfering with RIPK2 ubiquitylation^45^. This suggests a complex regulatory network of autophagy and NLR innate immune pathway regulation and future research is needed to clarify these differences.

The induction of selective autophagy by NLR proteins is not limited to bacterially induced NOD1/2 activation. Influenza A virus infection and cytosolic delivery of the dsRNA mimetic poly (I:C) also can activate NOD2 and RIPK2 dependent mitophagy via Ulk1 activation^45^. This mechanism may reduce immunopathology upon influenza A infection by dampening activation of the NLRP3 inflammasome^45^. This suggests a functional link between autophagy and the suppression of immunopathology. NAIP proteins, originally identified as susceptibility genes for *Legionella* infection, can target *Legionella pneumophila* containing vacuoles for autophagy^46^. NAIP proteins form an inflammasome with the NLR protein NLRC4 whereby NAIPs act as sensors for flagellin and components of the bacterial type III secretion system. Functionally, the NLRC4 inflammasome can induce turnover of LC3 at *L. pneumophila*-containing vesicles to control bacterial growth and prevent pyroptotic cell death to optimize anti-bacterial activity^47^.

### C.2. Mitophagy and NLR activation

Mitochondrial damage induced by pathogens is a general cellular mechanism. The NLRP3 and NLRC4 inflammasomes can sense this type of danger, resulting in the release of key inflammatory mediators IL-1β, and IL-18 and induction of pyroptosis (see below). NLRP3 and NLRC4 activation in macrophages is partly driven by mitochondrial damage and the release of mitochondrial DNA (mtDNA). Autophagy controls the magnitude of this response by sequestering mitochondria and mtDNA. Accordingly, LC3 deficiency leads to enhanced NLRP3-dependent LPS mortality in mice^48^ and intracellular *Pseudomonas aeruginosa* infection activates NLRC4 inflammasomes due to the cytosolic release of mtDNA, which is dampened by autophagy^49^. Of all human NLRs, only NLRX1 is predominantly localized at mitochondria and has several roles in controlling inflammatory and interferon responses as well as autophagy^50^. NLRX1 contains an LC3-interacting region (LIR) and interacts with LC3B. Both NLRX1 and LIR are important for *Listeria monocytogenes* induced mitophagy. ^51^ NLRX1 can also downregulate type I interferons by induction of autophagy during viral infection by interaction with Tu translational elongation factor mitochondrial (TUFM)^53^. Of note, NLRX1-mediated autophagosomal degradation of interferon pathway components including STING can contribute to immune evasion of papillomavirus transduced cancer cells^54^. By contrast, the interaction of NLRX1 with BECLIN1 was associated with negative regulation of autophagy of group A streptococci^55^.

### D. The connections between autophagy and inflammasome activity

The recognition of PAMPs by PRRs bearing cells such as macrophages triggers the innate immune system including inflammasomes. Inflammasomes are cytosolic signaling complexes that induce inflammation and can activate pyroptotic cell death. They consist of an NLR protein, the adaptor ASC and recruit pro-caspase 1 for autocatalytic activation^56,57^. The most extensively investigated canonical inflammasome uses NLRP3 as a sensor. NLRP3 serves to recognize PAMPs and host-derived damage-associated molecular pattern molecules (DAMPs). NLRP3 inflammasomes require two signals for activation, a priming signal that depends upon NF-κB, and a second signal provided by the inflammasome activator that triggers the assembly of NLRP3 with ASC, and caspase-1. This leads to the cleavage of pro-IL-1β and the release of mature IL-1β and IL-18. Crystal and cryo-EM structures of NLRC4 and a cryo-EM structure of the NLRP3-NEK7 complex have provided insights into the mechanism of inflammasome activation^58–61^. Non-canonical inflammasomes depend on caspase-11 (in mice) or caspase-4/5 (in humans)^62^. These inflammatory caspases sense intracellular LPS derived from Gram-negative bacteria and together with caspase-1 cleave the pore-forming protein gasdermin-D (GSDMD), which is needed for the cellular release of IL-1 cytokines but also permeabilizes the cell membrane and can trigger pyroptosis. GSDMD pore formation alters ion fluxes and can trigger NLRP3 inflammasome activation. Several recent reviews describe the molecular mechanisms underlying classical and non-classical inflammasome activation^63,64^, which is not discussed here.

Autophagy and the innate immune system are functionally intertwined. The finding that upon challenge, mouse macrophages lacking an essential autophagy component, ATG16L1, produced excessive amounts of the inflammasome-derived cytokines IL-1β and IL-18 provided early evidence for this connection^65^. Loss of ATG16L1 in hematopoietic cells also rendered mice highly susceptible to dextran sulfate sodium-induced acute colitis, an animal model of inflammatory bowel disease^65^. Upon activation, NLRP3 gets ubiquitinated and can recruit the autophagic adaptor p62/SQSTM1, leading to autophagic degradation of the inflammasome, which might be a central mechanism to end inflammasome activation in some cells^66^(Figure 2). Subsequent studies have revealed multiple interactions between the pathways that lead to autophagy and those that direct inflammasome assembly. A major mechanism is the autophagic removal of endogenous or exogenous inflammasome activators, thereby reducing the triggers for subsequent inflammasome activity. For example, the ubiquitin (Ub) sensor p62/SQSTM1 binds poly-Ub chains and can direct the removal of damaged mitochondria, which reduces NLRP3 inflammasome activation^67^. If autophagy is impaired, damaged mitochondria accumulate, which increases ROS production, mitochondrial DNA release, and subsequent NLRP3 inflammasome assembly^68^. The autophagy regulatory protein TRIM20 interacts with CASP1, NLRP1, and NLRP3 targeting them for degradation, hence preventing excessive IL1β- and IL18-mediated inflammation^69^ (Figure 2). TRIM20, encoded by the *MEFV* gene, a known risk locus for the autoinflammatory disease Familial Mediterranean Fever. The autophagy regulatory protein IRGM, which is upregulated in response to PAMPs and DAMPs, suppresses NLRP3 inflammasome activation by inhibiting its assembly. IRGM also mediates selective autophagic degradation of NLRP3 and ASC^70^, ^71^(Figure 2). Supporting human studies, a mouse ortholog of IRGM (Irgm1) limits gut inflammation in a mouse model of Crohn’s disease^70^. TLR4 stimulation of macrophages induces autophagy sequestering pro-IL-1β into autophagosomes leading to its degradation^72^. AIM2 inflammasomes can also be degraded by ubiquitin-dependent autophagy^73^ (Figure 2).

Several studies describe signaling events that regulate both inflammasomes and autophagy. The protein kinase WNK1 inhibits autophagy and also limits IL-1β production following NLRP3 inflammasome stimulation^74^. Depletion of WNK1 stimulates class III phosphatidylinositol 3-kinase complex (PI3KC3) activity, which induces autophagy^75^, however, a signaling pathway initiated by WNK1 balances intracellular ion concentrations during NLRP3 activation. In macrophages, its absence causes intracellular K^+^ and Cl^–^ levels to excessively decline, which augments macrophage IL-1β production^74^. Stimulation of the PI3K/AKT/mTOR pathway in macrophages, which often occurs following pathogen encounters, can limit both autophagy and inflammasome activation^76^. Upon activation of this pathway, mTORC1 inhibits ULK1, which phosphorylates the autophagy initiation machinery. AKT can also phosphorylate the N-terminus of NLRP3, limiting its oligomerization and reducing activation^77^. A known risk factor for Crohn’s disease, leprosy, and certain types of cancers, autosomal dominant mutations in *LRRK2* are the most common genetic cause of familial Parkinson’s disease^78^. In mice, *Lrrk2* deficiency reduces macrophage caspase-1 activation and IL-1β secretion in response to NLRC4 inflammasome activators. Lrrk2-deficient mice poorly clear *Salmonella* Typhimurium infections and they exhibit a marked impairment in selective forms of autophagy and lysosomal function; however, only a minor defect in nonselective autophagy^79^. Understanding the multiple regulatory mechanisms that control autophagy and inflammasomes may help design therapies to counter their manipulation by pathogens.

While initial studies largely focused on autophagy and inflammasomes in the setting of intracellular bacterial infection, the regulation and dysregulation of autophagy and inflammasomes contribute to the pathogenesis of many viral infections^80–82^. Autophagy can eliminate invading viruses and foster antiviral responses; however, some enveloped viruses use autophagy-related vesicles for transit and as sites for replication. SARS-CoV-2 has a complex effect on both autophagy and inflammasomes, and their dysregulation may contribute to the pathogenesis of severe, life-threatening diseases^83–85^. Several of the SARS CoV-2 encoded proteins manipulate autophagy. E protein, M protein, (open reading frame) ORF3a, and ORF7a all cause an accumulation of autophagosomes, whereas Nsp15 prevents their efficient formation. The viroporin ORF3a is a small, hydrophobic molecule that targets the host cell membrane altering their ion permeability^86^. ORF3a localizes to intracellular vesicles, in the endoplasmic reticulum, and at the plasma membrane. It helps viral egress via a lysosomal exocytosis-pathway^87,88^. Conversely, it induces a specialized form of autophagy termed reticulophagy through the high mobility group box 1 (HMGB1)-BECLIN1 pathway^89^. Inflammation releases nuclear HMGB1 into the cytosol and extracellular spaces where it helps sustain autophagy and functions as a DAMP. By disrupting ER homeostasis ORF3a induces ER stress and triggers inflammation. Evidence of inflammasome activation in COVID-19 patients has come from studies showing inflammasome ASC specks, active caspase-1, and cleaved GSMD in SARS-CoV-2 bearing monocytes ^90^. Cytokine release, immune cell recruitment, and positive feedback loops help drive the formation of the highly inflammatory milieu found in critically ill patients. Besides ORF3a the SARS-CoV-2 N protein also activates inflammasomes^91^. It directly interacts with NLRP3 promoting binding to ASC and facilitating NLRP3 inflammasome assembly. These and other data indicate that SARS-CoV-2 manipulates the host’s innate immune responses and that targeting these pathways should provide avenues to attenuate viral pathogenicity.

The central role of autophagy in inflammasome regulation is well supported by the fact that some bacterial pathogens manipulate the autophagy response as a subversion strategy. The SpvC effector protein from Salmonella blocks NLRP3 and NLRC4-induced autophagy^92^. In contrast, VopQ from *Vibrio parahaemolyticus* induces autophagy to dampen NLRC4 inflammasome activation^93^. *Shigella flexneri* which activates the NLRC4 inflammasome in macrophages can also induce autophagy to reduce IL-1β release and pyroptotic cell death^94^ and *Mycobacterium tuberculosis*-induced IL-1β release is also negatively controlled by autophagy^95^.

The autophagy-dependent regulation of inflammasome responses might be of use for therapeutic intervention for the treatment of hereditary disorders associated with mutations in NLRs. Rapamycin for example was shown to reduce NLRC4-mediated IL-1 cytokine production in myeloid cells expressing a disease-associated hypermorph of NLRC4^96^. Future research will help to delineate the whole picture of NLR-autophagy crosstalk. Emergent studies suggest that besides NOD1/2, NLRP3 and NLRC4, several other NLR proteins are linked to autophagy. The MHC class I regulator NLRC5, for example, was shown to be degraded by autophagy in endometrial cancer cells, leading to loss of MHC class I expression that was associated with worse clinical outcomes^97^. Besides the NLR discussed above, NLRC4, NLRP3, NLRP4, and NLRP10 were also shown to interact with BECLIN1 and for NLRP4 it was shown that recruitment to *Streptococci* containing phagosomes led to the release of BECLIN1 resulting in the local induction of autophagy^98^. In the gut, NLRP6 regulates autophagy in goblet cells to control mucus production^99^. Taken together, this suggests the potential of NLR proteins as targets to tailor inflammophagy responses and in line with the recent characterization of specific inhibitors for RIPK2 and NLRP3 has clinical potential.

### E. Interplay between RLRs and Autophagy

Another family of intracellular PRRs is the RIG-I-like receptors (RLRs). The three ubiquitously expressed RLR members are RIG-I, MDA5, and LGP2 in contrast to the NLRs described above mainly act in anti-viral responses. Upon activation via cytoplasmic viral RNA or processed self RNA, RLRs initiate a series of events that activate interferon regulatory factors (IRFs)^100,101^. IRFs are the key transcription regulators of Interferons (mainly type I and type III IFNs).

RIG-I and MDA5 are the most studied members of the RLR family. Upon sensing PAMPs, RIG-I oligomerizes and interacts with adaptor protein mitochondrial antiviral signaling (MAVS) on the mitochondria. MAVS oligomerizes to form large aggregates and associates with multiple adaptor proteins including TRAF2, TRAF3 TRAF6, TANK, and TRADD to activate Tank-binding kinase-1 (TBK1) and I kappa B kinase epsilon (IKKε)^100,101^. The IRFs (IRF3/7) are phosphorylated by activated TBK1 and IKKε resulting in their homo- or heterodimerization and translocation to the nucleus to promote the expression of IFNs. The secreted interferons are sensed by interferon receptors leading to the activation of JAK-STAT1/2 pathways resulting in the upregulation of a large number of interferon-stimulated genes (ISGs) that have a myriad of functions in innate immunity^100,101^.

The interplay between RIG-I signaling and autophagy has been documented, however, mechanistically, how RIG-I signaling participates in autophagy modulation is not very clear. On the other hand, how autophagy suppresses RIG-I-MAVS signaling is well understood. The activation of RIG-I-MAVS-TRAF6 signaling by PAMPs and virus induces the interaction between TRAF6 and BECLIN1 resulting in increased K63-linked ubiquitination of BECLIN1^102^, which is an important event in autophagy upregulation (Figure 1).

Several autophagy-dependent mechanisms target RIG-I, MDA5, and MAVS for degradation to suppress inflammation. In the absence of autophagy, dysfunctional mitochondria accumulate leading to the production of high levels of mtROS that likely activates RLR signaling leading to IFN production^103^. The Atg5–Atg12 conjugate can directly interact with the CARD domains of MAVS and RIG-I to suppress the production of type I IFN’s^104^. Upon viral cellular invasion, the leucine-rich repeat-containing protein 25 (LRRC25) interacts with activated ISG15-tagged RIG-I and enhances its interaction with autophagy receptor p62/SQSTM1^105^. The ISG15-tagged RIG-I is delivered to the autophagosome via p62/SQSTM1 for degradation suppressing type I IFN signaling (Figure 2). Recently, the coiled-coil domain containing 50 (CCDC50) protein was found to be a new receptor for autophagic degradation of K63-polyubiquitinated RIG-I/MDA5^106^ (Figure 2). CCDC50 directly interacts with LC3 protein on autophagosome membranes to deliver ubiquitinated RIG-I/MDA5, thereby suppressing the type 1 IFN response. CCDC50 deficient mice exhibited a reduced autophagic degradation of RIG-I/MDA5, a heightened type I IFN response, and enhanced viral resistance^106^.

MAVS is targeted by several proteins including Tetherin, RNF34, and HFE for autophagic degradation^107–109^ (Figure 2). Tetherin, an ISG and anti-viral protein that recruits MARCH8 to enhance K27-linked ubiquitination of MAVS, is recognized by autophagy receptor protein NDP52^107^. NDP52 mediates autophagic degradation of MAVS and suppression of type I IFN response. Similarly, RNF34, a ring finger domain-containing E3-ligase, interacts and catalyzes the K27 and K29-linked ubiquitination of MAVS for NDP52-dependent autophagy resulting in reduced type I IFN responses^108^.

Several studies have shown genetic and functional linkage of IRGM protein with type 1 interferonopathies and autoimmune diseases^25,110,111^. The mechanism remained unclear until recently when IRGM was shown to directly interact with RIG-I and MAVS to mediate RIG-I degradation via p62/SQSTM1-dependent selective autophagy (Figure 2). IRGM strongly suppresses type 1 IFN response and reducing its expression induces more than 100 ISGs^25^. Similar results were obtained in Irgm1-deficient mice. Downregulation of IRGM/Irgm1 in humans and mice causes mitophagy defects^25,110^. The resulting accumulation of defunct mitochondria increased mtROS, mtRNA, and mtDNA fueled RIG-I-MAVS and cGAS-STING signaling for the production of IFN response. These studies delineated the mechanisms by which IRGM protects against type 1 interferonopathies and autoinflammatory diseases. On the flip side, since IRGM is a master negative regulator of the interferon response, its depletion could suppress the replication of a large number of different RNA/DNA viruses^112^.

Thus, autophagy-dependent mechanisms play a vital role in the suppression of RLRs mediated cytokine response and hence maintain innate immune balance during microbial infection or sterile damage.

### F. cGAS-STING signaling and autophagy

Cyclic GMP-AMP synthase (cGAS) senses cytosolic dsDNA to trigger activation of STING (TMEM173) protein to induce both type I interferon and NF-κB responses^113^. Extensive functional interactions have been documented between autophagy and the cGAS-STING pathway. Stimulation of the cGAS-STING pathway not only induces type 1 IFN response but also enhances autophagy by several mechanisms to suppress IFN response and thus maintain immune homeostasis. For example, cGAS interaction with autophagy protein BECLIN1 results in reduced cGAMP synthesis and enhanced autophagy-dependent degradation of dsDNA causing suppression of the IFN response^114^. Genotoxic stress results in increased micronuclei in the cytosol that are suggested to be sensed by cGAS and induce interferon response. Recently, it was shown that cGAS also acts as a receptor for micronuclei and could target them for autophagy by directly interacting with LC3B on autophagosomes^115^. By doing so, cGAS maintains the balance of the IFN response induced against micronuclei.

STING can induce LC3 lipidation and upregulation of autophagy (Figure 1). The cGAMP-induced STING-containing ERGIC (Endoplasmic reticulum–Golgi intermediate compartment) provides a membrane source for LC3 lipidation and autophagosome biogenesis^116^. The cGAMP-induced STING-dependent autophagy could clear DNA and viruses from the cytoplasm, possibly to reduces the source of inflammation. Another study confirmed STING-induced LC3 lipidation but reported that LC3 lipidation occurs at single membrane perinuclear vesicles and is mediated by v-ATPase and ATG16L1^117^.

cGAS and STING themselves are degraded by p62/SQSTM1-dependent autophagy to maintain the homeostatic balance of the IFN response^25,118^(Figure 2). Interestingly, STING-activated TBK1 can phosphorylate both IRF3 and p62/SQSTM1. Activated IRF3 induces type I IFN production, whereas phosphorylated p62/SQSTM1 interacts with STING to degrade it and suppress type I IFN response^118^. IRGM interacts with cGAS to enhance p62/SQSTM1-dependent autophagic degradation resulting in suppression of type I interferon response^25^. Altogether, the cGAS-STING pathway like NLR, TLR, and RLR signaling can induce autophagy and in most cases, this autophagy can suppress inflammation in a feedback loop.

### G. Complement-mediated modulation of autophagy

Complement is a tightly regulated system that critically contributes to the humoral innate immune defense against pathogens and promotes local inflammation, phagocytosis, and lysis of invading microbes. Several components of the complement system can be involved in anti-microbial autophagy (Figure 1). Attenuated measles virus (MeV) infects human cells through CD46, a regulatory factor that prevents complement-mediated cell lysis. MeV entry rapidly activates autophagy through the CD46-Cyt-1 intracytoplasmic splice variant that recruits the scaffold protein GOPC, an interactor of the VPS34-BECLIN1 complex^119^. This early autophagy resolves spontaneously before a second and sustained autophagic phase that depends on virus replication and benefits from it^120^. The CD46-Cyt-1-GOPC-VPS34-BECN1 axis also activates autophagy during infection by *Group A Streptococcus* (GAS)^119^ and *Neisseria gonorrhoeae*^121^, both bind to CD46. Because CD46 also serves as a receptor for other viruses (HHV-6, BVDV pestivirus, and adenoviruses B/D), this axis is likely to activate autophagy upon infection by additional pathogens. Thus, the complement regulatory factor CD46 acts as a pathogen sensor able to initiate autophagy upon infection (Figure 1). V-set and immunoglobulin domain containing 4 (VSIG4) is a surface receptor expressed on phagocytic antigen-presenting cells and functions as a receptor for the phagocytosis of bacteria opsonized with the complement factor C3. VSIG4 engagement by C3b-coated *Listeria monocytogenes* promotes the ubiquitination and targeting of cytosolic bacteria to autophagic degradation in both macrophage-like cells and primary macrophages^122^. Hence, by engaging the VSIG4 receptor, C3b deposited on *L. monocytogenes* induces an antibacterial autophagic response in professional phagocytic cells. C3b deposited on *L. monocytogenes* or adherent-invasive *Escherichia coli* (AIEC), can also activate anti-microbial autophagy in epithelial cells after reaching the cytosol^123^ (Figure 1). This capacity relies on the direct interaction of C3 with ATG16L1 and is altered in cells lacking ATG16L1 or carrying its T300A variant that is associated with Crohn’s disease. The strong anti-microbial potential of this effect is best illustrated by the fact that some bacteria evolved strategies to counteract the C3-ATG16L1 interaction^124^. For instance, opsonized *Shigella flexneri* and *S. typhimurium* can attenuate autophagy restriction through the shedding of adsorbed C3 soon after infection by engaging proteases of the omptin family^124^. These studies thus indicate that complement factors can regulate the autophagic response to intracellular microbes through both surface and cytosolic interactions.

## 2. Interferon-inducible GTPases (IRGs) and autophagy

The Immunity-Related GTPases (IRG; also known as p47 GTPases) and the Guanylate Binding Proteins (GBP, also known as p65 GTPases) are related families of GTPases whose expression is dramatically induced by type I (IFN α/β) and type II (IFNγ) interferons^125,126^. The genes encoding these GTPases were cloned beginning in the late 1990’s as a consequence of genetic searches for IFN-induced genes. The existence of GBP proteins was actually known earlier, as their high levels in cell lysates from IFN-induced cells result in intense, diagnostic spots on two-dimensional protein gels. IRG genes are abundant in rodents with 23 family members found in C57BL/6 mice^127^. In contrast, IRGs are more restricted in humans where there are two members - IRGC, which is constitutively expressed only in the testis, and IRGM that is more widely expressed but truncated relative to mouse IRGs^127^. Genes encoding IRGs are present in small numbers in other species such as dogs, fish etc.,^127,128^. These patterns suggest that IRGs have proliferated in rodents under evolutionary pressure from endemic pathogens and play more expansive roles in rodent innate immunity, though important roles in humans exist. The GBP gene families are more equitable between rodents and humans, perhaps suggesting a higher degree of functional conservation.

IRGs/GBPs are expressed in a range of hematopoietic and non-hematopoietic cells following IFN activation. Their activities are essential for normal IFN-induced immune responses to some pathogens^129–131^. IRGs and GBPs are thought to function as dynamins, a superfamily of large GTPases that bind cellular membranes and then undergo GTP hydrolysis that induces conformational changes and enables various cellular functions^132^. For instance, in Irgb6 conformational changes following GTP hydrolysis allow recognition of a phospholipid binding site on the vacuolar membranes surrounding *Toxoplasma gondii*^133^. IRGs and GBPs bind diverse membrane compartments where they are thought to carry out a collection of activities that support two major cellular functions: cell-autonomous immunity and autophagic regulation.

### A. IRGs/GBPs as Regulators of Autophagy of Cell Autonomous Immunity

**Figure 3.**
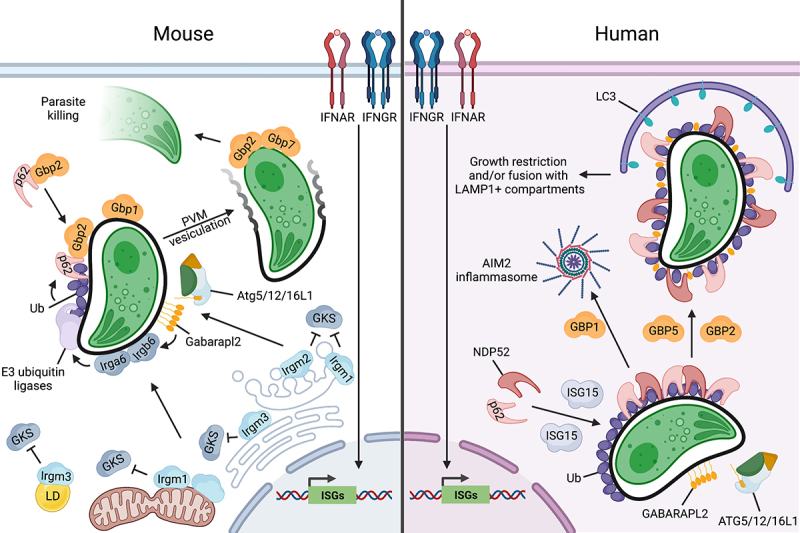
Mechanisms of cell-autonomous immunity to *Toxoplasma* in mouse and human cells. Mouse: Activation of IFNAR or IFNGR by type I or II IFNs, respectively, induces expression of >1000 interferon-stimulated genes (ISGs), including IRGs and GBPs. Irgm proteins reside on host organelles including Golgi (Irgm1/m2), ER (Irgm3), mitochondria (Irgm1), and lipid droplets (LDs; Irgm3) and inhibit the binding and activation of cytosolic GKS proteins on those host membranes. Irgm2 promotes the conjugation of GABARAPL2 on the PVM, which promotes GKS and GBP recruitment. Irgm1 and Irgm3 coordinate the targeting of GKS proteins to the *Toxoplasma* PV, with Irgb6 acting as a pioneer to recruit other GKS proteins such as Irga6. GKS targeting to the PV promotes PV ubiquitination and ubiquitin-dependent recruitment of cytosolic complexes containing p62/SQSTM1 and GBP2, which in turn promotes recruitment of other GBPs including GBP1. Decoration of the PV with host effectors leads to vesiculation of the PVM, and the exposed *Toxoplasma* is targeted by GBPs including GBP2 and GBP7, and destroyed. **Human**: IFN stimulation drives the expression of ISGs including GBPs and ISG15 as well as ubiquitination of the *Toxoplasma* PVM. ISG15 promotes the recruitment of p62/SQSTM1 and NDP52 to ubiquitinated PVs, which facilitates association with LC3+ membrane structures. In some cell types, this leads to restriction of *Toxoplasma* growth and replication, and in others, the PV is delivered to LAMP1+ lysosomes for degradation. Decoration of the PVM with GABARAPL2 drives parasite clearance by an unknown mechanism. GBP1 is recruited to the *Toxoplasma* PV in macrophages but not A549 cells and liberates parasite DNA for activation of the AIM2 inflammasome. GBP2 and GBP5 are not recruited to the PV but contribute to the clearance of *Toxoplasma*.

The first immune function assigned to mouse IRGs was the ability to kill *Toxoplasma gondii* and *Chlamydia trachomatis* in host cells (macrophages, astrocytes, and fibroblasts) that have been activated with IFN^134–139^. A large family of over 20 mouse IRGs bifurcates into two types: the majority are known as GKS IRGs because they possess a canonical GKS sequence motif in the GTP-binding region, while the less common GMS or IRGM proteins possess a non-canonical GMS motif^128^. The GKS IRGs are the major players in this cell-autonomous killing mechanism. They are present in the cytoplasm or on membranes in host cells, but following *T. gondii* or *C. trachomatis* infection, they rapidly home to the parasitophorous vacuole (PV) that surrounds the pathogens^140–138–142^. They do so in a hierarchical order that ostensibly is required for function, with Irgb6 being a lead protein that recognizes specific phospholipids on the *T. gondii* PV^143^ (Figure 3). Once the GKS IRG complex coats the PV, the *T. gondii* PV vesiculates extruding the naked pathogen into the cytoplasm, effectively stripping the parasite of its protective niche and leading to its death^136,144^ (Figure 3). This vesiculation function is reminiscent of the ability of classical dynamins to contort membranes to form vesicles. The GKS-coated *C. trachomatis* PVs similarly undergo lytic disintegration^145^, suggesting a conserved host defense mechanism executed by mouse IRGs. The mouse IRG family is highly diverse among wild species of mice and has likely been shaped by co-evolution with pathogens like *T. gondii* that naturally infect rodents^146^. IRGM proteins play a more peripheral role in these processes, as they bind endomembranes in the cell and hold the GKS IRGs in a biochemically inactive state in those locations until they are needed to target PVs^141,147^. GMS proteins thus play a critical chaperone role and in their absence, the GKS proteins aggregate and lose their capacity to correctly target to pathogen membranes^141,148,149^. The central role of this cell-autonomous killing mechanism for pathogen control is illustrated by the fact that *T. gondii* and *C. muridarum*, a rodent-adapted pathogen, have both evolved the capacity to circumvent IRG-mediated killing. In *T. gondii*, specific virulence factors (rhoptry proteins ROP5, ROP17, and ROP18) phosphorylate GKS IRGs to inactivate them^150–152^. This ability to neutralize IRGs, or not, determines the overall virulence category of *T. gondii* stains. *C. muridarum* can also avoid GKS-mediated killing^142^ although the molecular basis for this evasion is not yet defined.

GBPs also target *T. gondii* PV and *C. trachomatis* inclusion. The GBP family in mice consists of two clusters one on Chromosome (Chr) 3 (containing Gbp1. Gbp2, Gbp3, Gbp5, Gbp7, and Gbp2ps) and one on Chr 5 (containing Gbp4, Gbp6, Gbp8-11)^153^. Deletion of the Chr3 cluster renders mice susceptible to infection with *T. gondii*^153^. Like IRGs, GBPs also home to PVs in an ordered manner with Gbp1^154^ and Gbp2^155^ initially recruited to the PV surrounding *T. gondii*, while Gbp2 and Gbp7 are thought to target the parasite membrane for destruction after the vacuole has ruptured^156^ (Figure 3). In contrast to IRGs, GBPs may not affect PV vesiculation of *C. trachomatis* PVs; rather, they are involved in inflammasome activation^145,157^. There is robust cross-regulation among the GBPs and the two IRG subfamilies: IRGM proteins can influence the recruitment of GBPs to PVs^158^, and GBPs can affect the recruitment of IRGs^159,160^. Ubiquitination by the E3 ubiquitin ligase TRAF6 and the autophagy adapter protein p62/SQSTM1is also involved in the process by recruiting GBPs to the PV^145^ (Figure 3).

Although autophagy is not required for vesiculation of PVs per se, autophagy and IRGs/ GBPs do cooperate to recognize and eradicate *T. gondii* in a cell-autonomous manner in murine cells^161–163^. This process does not require the initiation steps of autophagy (i.e. Beclin and Atg14) nor the degradative steps (lysosomal fusion), but rather relies on a core set of ATG proteins that has been referred to as non-canonical autophagy^164–166^. The autophagy proteins Atg3, Atg7, and the Atg5-Atg12-Atg16L1 complex are recruited directly to the PV, perhaps by recognition of phosphatidylinositols in the PV membrane^143,163^(Figure 3). This non-canonical autophagy protein complex then conjugates LC3 homologs (Atg8 and the GABARAPs) to phosphatidylethanolamine in the membrane. Of the five LC3 orthologs in mice, the major effector is Gabarapl2, while others play lessor roles^167^(Figure 3). It is thought that the recruitment of this ATG complex to the PV membrane facilitates the recruitment of IRGs/ GBPs that subsequently carry out vacuole lysis. In the absence of a core set of ATG proteins (i.e. Atg3, Atg7, Atg5-Atg12-Atg16), IRGs and GBPs become activated and aggregate in cytoplasmic clusters and are therefore unable to function in recruitment to pathogen containing vacuoles^165,168^(Figure 3). The lack of ATG proteins thus leads to an absence of IRG/ GBP homing to the PV, essentially undermining the whole cell-autonomous mechanism in mouse cells.

In contrast to murine cells, where GKS IRGs play a prominent role, human cells lack this set of effectors and yet still control *T. gondii* through a process that relies on non-canonical autophagy and GBPs. Human cells express seven GBPs that are found in a single cluster on Chr1^169^. It should be noted that in human cells autophagy proteins are dispensable for GBP delivery but essential for the recruitment of GABARAPL2^170^(Figure 3), which is required for cell-autonomous restriction of *T. gondii*, though presumably utilizing different effector proteins^168,170^ In HeLa cells, the process is initiated by ubiquitination of unknown targets on the PV followed by recruitment of adaptors p62/SQSTM1, NDP52 and finally conjugation of LC3^168^. Unlike the process of xenophagy that restricts intracellular bacteria^171^, control of *T. gondii* in human cells by noncanonical autophagy requires activation with IFN-γ. Autophagy proteins are not normally induced by IFN- γ, rather the link between these two pathways is mediated by induction of ISG15, which facilitates recruitment of the Ub binding adaptors p62/SQSTM1 and NDP52 to the PV surrounding *T. gondii*^172^. The fate of LC3 positive vacuoles differs somewhat based on cell type: in HeLa cells engulfment in LC3 positive membranes stunts parasite growth^168^, while in HUVEC cells the parasite is delivered to LAMP1+ compartments^173^. In human macrophages, GBP1 performs its role following recruitment to the PV ^174^, although it acts at a distance in A549 human lung epithelial cells infected with the protists *T. gondii* or *Leishmania donovani*^175,176^. GBP1 also contributes to parasite restriction by inducing the AIM2 inflammasome following the release of parasite DNA into the cytosol^177^(Figure 3). Additionally, GBP2 and GBP5 have been implicated in parasite control despite not being recruited to the vacuole^178,179^(Figure 3). Both the GTPase functions and lipidation are required for the activities of GBP1, GBP2, and GBP 5 in human monocytes treated with IFN- γ^178^. The ability of GBPs to act at a distance contrasts with the role of such effectors normally have in targeting the PV membrane or parasite within.^177^.

Beyond the vesiculation mechanism, IRGs and GBPs can recruit other anti-microbial factors that function on a cell-autonomous level. These factors are numerous and once recruited to vacuoles/phagosomes or bacteria, they act through diverse mechanisms. For instance, Irgm1 and Irgm3 control the translocation of multiple E3 ubiquitin ligases including TRAF6 to *T. gondii* and *C. trachomatis* vacuoles^145,180^, which then tags the vacuoles with ubiquitin to allow recognition by other cellular factors. In contrast, Irgm2 controls the targeting of Gabarapl2 to *T. gondii* phagosomes^181^, which then modifies phagosome processing. Regarding GBPs, they have been reported to control the targeting of phagocyte oxidase, antimicrobial peptides, and autophagy effectors to membrane-bound intracellular bacteria to elicit their killing^131^. Further, GBP complexes assemble on cytosolic bacteria, where they can recruit caspases that trigger inflammasome activation^182,183^. GBPs are also able to block actin tail-mediated motility of cytosolic bacteria such as Shigella^183–186^. Finally, both GBPs and IRGMs modulate the sensing of cytosolic LPS^187–190^. The underlying biochemical mechanisms are not yet clear that allow IRGs/ GBPs to recruit these factors; nor it is clear how these activities hinge on the core dynamin-like activity of the proteins.

### B. IRGM as Regulators of Autophagy

IRG/GBP proteins regulate autophagic processes that are important for pathogen control and limiting potentially excessive inflammatory cytokine production. This is underscored by polymorphisms in the human IRGM gene that reduce IRGM expression and autophagic activity, and are associated with Crohn’s Disease^191,192^, ankylosing spondylitis^193^, non-alcoholic fatty liver disease^194^, and *Mycobacterium tuberculosis* infection^195^, as well as poor outcomes to sepsis^196^. IRGM has been shown to regulate the assembly of the core autophagic machinery^42^. It does so by interacting with ULK1 and BECLIN1 to promote their assembly into autophagy initiation complexes. IRGM also interacts with NOD2, which enhances the K63-linked polyubiquitination of IRGM that is required for interactions of the protein with the autophagy complex^42^. This ability to promote autophagy impacts immunity and inflammation in various ways. For instance, mouse Irgm1 and human IRGM both stimulate autophagic killing of phagosomal *M. tuberculosis*^197,198^ and adherent-invasive *Escherichia coli*^199^ in macrophages. Both proteins regulate autophagic degradation of NLRP3 and ASC, which has the effect of blocking NLRP3 inflammasome activation and limiting IL-1β production^70^. IRGM also targets viral replication complexes that are subsequently ubiquitinated and thus tagged for autophagic removal^200^.

A particularly important aspect of the involvement of Irgm1/IRGM in autophagy may be in their ability to promote autophagic clearance of mitochondria, a process known as mitophagy. When Irgm1 and/or IRGM is absent, mitochondrial homeostasis is perturbed as reflected in an accumulation of defective mitochondria, altered mitochondrial fission/fusion states, and increased presence of intracellular mitochondrial DNA in the cytoplasm^24,25,201–204^. These alterations in mitochondria result in changes in cellular metabolism that shape immune cell function and increase inflammation^25,205^. The mitochondrial DNA/RNA soiling of the cytoplasm has profound consequences, as it activates intracellular sensors such as cGAS/STING. RIG-I-MAVS, and TLR7 that trigger a cascade of cytokine production^24,25,204^. This notably drives a type I ‘interferonopathy’ that significantly modulates multiple immune responses and is a key driver of disease.

## 3. Autophagy and adaptive immunity

### A. Autophagy and MHC class II antigen presentation

Because class II molecules of the major histocompatibility complex (MHC II) constitutively traffic through endo-lysosomal acidic compartments where they get complexed to antigenic peptides (MHC II compartments), autophagosomal cargoes that cross such compartments can influence MHC class II antigen presentation to CD4+ T lymphocytes helper cells. This happens in various cell types such as epithelial cells, melanocytes, and professional antigen-presenting cells, including B cells^206–210^. As a consequence, conjugating antigens to LC3 promotes their MHC II presentation due to imposed autophagosomal targeting^210–214,215^. Autophagy can promote the MHC II presentation of endogenous (self) antigens of both cytosolic and nuclear origin, including factors of the ATG8 family and autophagy receptors such as TAX1BP1^208, 216^. In the case of thymic epithelial cells (TECs)^217^, such an effect participates in the selection of the T cell receptor (TCR) repertoire for both effector and regulatory (Treg) CD4+CD8- thymocytes^211,218,219^. Accordingly, interfering with TEC autophagy can trigger multi-organ inflammation^218^, and fusing antigens to LC3 leads to the intra-thymic deletion of immature CD4+CD8- thymocytes expressing cognate TCRs^211^. The role of autophagy in the antigen-presenting function of TECs appears to be regulated by the C-type lectin CLEC16A whose deficiency diminishes the autophagic activity of TECs and attenuates the positive selection of autoreactive thymocytes^220^. How CLEC16A positively influences autophagy in TECs remains to be dissected as it can also repress autophagy in epithelial cells through activation of the mTOR pathway^221^. Autophagy also contributes to the MHC II presentation of extracellular antigens, including determinants derived from viruses and bacteria^209,207,222–226^. Indeed, the influence of autophagy on the MHC II presentation of microbial antigens can be so efficient that microorganisms evolved means to interfere with it^223–230^.

### B. Autophagy and MHC I antigen presentation

MHC class I molecules are specialized in the acquisition of antigenic peptides resulting from proteasomal degradation for presentation to CD8+ T lymphocytes. This acquisition occurs within the endoplasmic reticulum (ER) after the importation of cytosolic peptides. Autophagy appears to influence MHC I-peptide complexation when this classical MHC I pathway is perturbed for instance by viruses that interfere with peptide transport into the ER. In that context, some complexation events can take place in late endosomal compartments that receive contents from autophagosomes. This appears to be the case during infection with the herpes simplex virus (HSV) 1 or human cytomegalovirus (HCMV) for the MHC I presentation of peptides from HSVgB and pUL138 viral antigens, respectively^231,232^. However, autophagy negatively regulates the level of MHC I antigen presentation by promoting cell surface MHC I molecules internalization and lysosomal degradation and thereby, negatively modulating the presentation of viral or tumor antigens to CD8 T cells^233–235^. In fact, both MHC I and non-classical MHC I (such as CD1d) molecule expression is augmented on the surface of professional antigen-presenting cells lacking ATG5 or ATG7 factors^226,233^. In this context, the adaptor-associated kinase 1 (AAK1) which regulates the activity of the AP2 complex is not properly recruited to MHC I molecules^233^, indicating a role for autophagy factors in some forms of endocytosis. In tumor cells, MHC I degradation can involve the autophagy receptor NBR1 that mediates ERphagy^235^.

The ability of MHC I molecules to present peptides from exogenous antigens is named cross-presentation. This process, which is very efficient in some subsets of DCs, may depend on the persistence of internalized antigens in endosomes that intersect with recycling endosomes carrying MHC I molecules. In some instances, core autophagy genes may be required for such a phenomenon. Thus, Atg7 is needed for the MHC I cross-presentation of peptides from soluble ovalbumin (OVA) but not that of OVA from apoptotic cell corpses or OVA directed to the DEC205 endocytic receptor^236^. In B cells, the cross-presentation of protein antigens can require both autophagy and proteasome activity^237^. Cross presentation of some extracellular antigens on MHC I can be affected by the absence of VPS34^238^. On the donor cell side, functional autophagy can favor the cross-presentation of both viral and tumor antigens by efficiently conditioning exosomal vesicles captured by dendritic cells^239–241^. In addition, a contribution of autophagy to the transfer of endocytosed antigenic material into the cytosol before connection to the classical MHC I pathway has been suggested^242,243^, although the exact modalities of the autophagy machinery contribution to exocytosis remain to be elucidated. Hence, while the autophagy machinery limits the presentation of endogenous antigens by regulating MHC I expression level, it can facilitate MHC I presentation of some forms of exogenous antigens by acting both in presenting cells and antigen donor cells.

### C. Autophagy in T lymphocyte biology

**Figure 4.**
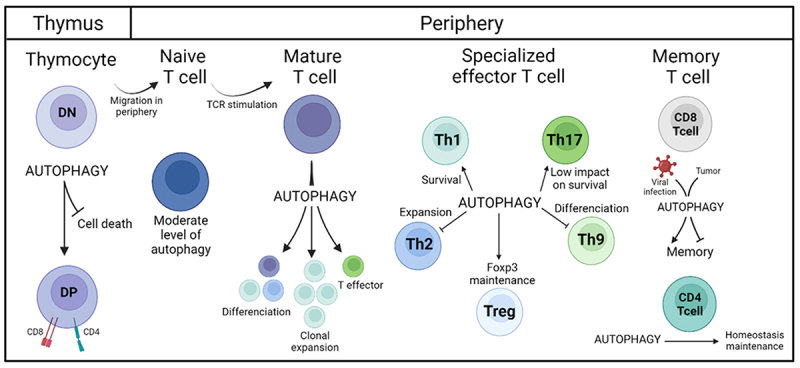
Autophagy in T lymphocyte biology. Autophagy is critical for T cell homeostasis during their development and their effector functions. In thymocytes, autophagy prevents cell death and allows the transition from double-negative (DN) CD4^–^CD8^–^ cells to double-positive (DP) CD4^+^CD8^+^ cells. After they migrate to the periphery, resting naïve T cells harbor a low level of autophagy. However, upon TCR engagement, autophagy is important for the differentiation, clonal expansion, and the effector function of mature T cells. Autophagy also has a role in the maintenance of memory T cells.

Autophagy is instrumental for T lymphocyte homeostasis both during development and their effector functions. It also has a role in immunological memory (Figure 4). Lymphocyte activation via antigen receptor (TCR) engagement triggers multiple biochemical changes that are necessary for clonal expansion and effector cell differentiation. Most notably, lymphocytes increase their glucose uptake, glycolytic activity, and glutamine metabolism to generate ATP and the metabolites required for activation and effector functions, a transition that involves contributions from autophagy. During thymic development, autophagy is important for the CD4-CD8- to CD4+CD8+ transition^244^ before commitment to either CD4+CD8- or CD4-CD8+ lineages and exit into the periphery (Figure 4). Mice lacking core autophagy factors, such as those involved in the initiation/conjugation steps, display a reduced pool of both immature and mature T cells due to increased cell death^245–247^ indicating an important role for autophagy in the homeostasis of both developing and mature T cells (Figure 4). Autophagy is also important for the development of invariant NKT cells, a specialized subset of T cells that react to microbial glycolipid in the context of CD1d^248^.

Naïve resting T cells possess a moderate level of constitutive autophagy and depend on oxidative phosphorylation for their energetic needs. Upon T cell activation, the overall autophagy flux is enhanced^245,246,249^ while the autophagic degradation of mitochondria is diminished^250,251^. During this process, autophagy protects T cells from a form of functional inactivation called anergy^252^. During differentiation into helper (CD4+) or cytotoxic (CD8+) T cell subsets, autophagic and metabolic interconnected adjustments influence the functional potential of the resulting cells. The proinflammatory Th1 subset of differentiated CD4 T cells is more dependent on autophagy than other subsets, such as Th17 cells, for their survival^253,254^(Figure 4). In Th2 cells, autophagy appears to negatively regulate their persistence as they further persist/expand in its absence^253,255^. Along the same line, autophagy deficiency favors the differentiation and function of Th9 CD4 T cells^256^. Thus, autophagy differentially impacts the differentiation of naïve CD4 T cells into their specialized effector subsets. In the absence of mTOR, CD4 T cell differentiation is skewed toward regulatory T cells (Treg), which are important for repressing autoimmune T cells, at the expense of conventional CD4 T helper cell subsets. Tregs rely on fatty acid oxidation for energy generation while effector CD4 T cells favor glycolysis to support their function^257–260^. Deficiency in ATG16L1, Vps34, ATG5, or ATG7 alters the persistence and function of Tregs while it promotes those of Th2 cells^255,261,262^ revealing the importance of autophagy in Treg maintenance, especially in terms of transcriptional reprogramming (Figure 4). Alteration of autophagy promotes mTOR activation and glycolytic enzyme expression, which enhances glycolysis and better fits the requirements of conventional T helper cells. In CD8 T cells, antigen receptor activation leads to augmented glycolysis but differs from CD4 T cells as there is a significant role for the pyruvate dehydrogenase^263^. Upon viral infection, the importance of functional autophagy in effector CD8 T cells varies according to the involved pathogen^264,265^. After contraction of the effector response, some CD8 T cells revert to oxidative phosphorylation and acquire a status of long-lived, antigen-specific, memory cells. As autophagy can be important for such metabolic adjustments it is not surprising that the emergence and persistence of CD8 memory T cells are greatly sensitive to the autophagy status^264–266^. For instance, CD8 T deficient in ATG7 after encountering viral antigens were altered in their capacity to generate a memory pool^264^, and autophagy was found to promote the maintenance of liver resident memory CD8 T cells^267^. In the context of tumor antigens, autophagy represses the function of effector/memory CD8 T cells by controlling histone methylation, glycolysis, and immune response gene expression^268^, Finally, autophagy can act positively on stemness and survival of antigen-experienced CD8 T cells within the tumor microenvironment^269^. In many situations, the metabolic profile of CD8 T cells was modified by the level of cell-autonomous autophagy confirming that autophagy-associated changes in metabolism greatly influence the generation and function of CD8 memory T cells (Figure 4). Within the CD4 T cell lineage, autophagy was found important for the homeostasis of lymphoid organ memory T cells by regulating the mitochondrial pool and lipid load^270^.

## 4. Non-canonical autophagy, infection, and immune response

Non-canonical autophagy has emerged as an essential component of the innate immune system, which serves as a cell-autonomous defense mechanism. Non-canonical autophagy regulates pathogen clearance, inflammation, and antigen presentation. Thus, it plays an indispensable role in protecting against infectious, autoimmune, and inflammatory diseases^271–273^.

Unlike the involvement of LC3 with double-membrane autophagosomes in macroautophagy, non-canonical autophagy involves conjugating LC3 family proteins to single-membrane compartments in a ULK1/2-independent manner. For example, LC3 conjugation to the phagosomal membrane (LAPosome) occurs by a process called LC3-associated phagocytosis (LAP), while LC3-associated endocytosis (LANDO) involves conjugation of LC3 to endosomes. Interestingly, Rubicon, a negative regulator of canonical autophagy, positively regulates non-canonical autophagy pathways. Apart from Rubicon, either BECLIN1-VSP34 complex generated PI3P, or PI3P independent LC3 conjugation on the single membrane, requires the involvement of canonical autophagy elongation complex (ATG12-ATG5-ATG16L1)^274–276^. The later process of covalent association of LC3 with a bilayer is known as the *C*onjugation of *A*TG8 to *S*ingle *M*embranes (CASM). During CASM, in addition to phosphatidylethanolamine (PE), LC3/GABARAP can also be conjugated to phosphatidylserine (PS)^277^. While similarities and differences exist between these two autophagy processes, we focus on the crosstalk between non-canonical autophagy (LAP/LANDO) and innate immunity to maintain immune homeostasis. We also highlight the unconventional roles of autophagy proteins beyond their role in autophagy, especially on innate immune regulation.

Douglas R. Green’s group first showed direct and rapid recruitment of LC3 on phagosomal membranes upon TLRs stimulation in mouse macrophages. This was shown to facilitate the maturation of phagosomes resulting in enhanced degradation of engulfed foreign entities during the early stages of infection^278^. The authors later named this process “LC3-associated phagocytosis (LAP)^279^. LAP has been implicated in the clearance of several intracellular pathogens such as *Listeria monocytogenes, Streptococcus pneumoniae, Aspergillus fumigatus, Salmonella typhimurium, Mycobacterium tuberculosis*, and Influenza A virus (IAV)^280–286^. As explained above for CASM, during LAP, LC3 lipidation to a single membrane requires the presence of PI3P, ROS, and ATG16L1. This occurs by the concerted action of Rubicon, NADPH oxidase (NOX), and V-ATPase. While Rubicon is important for the generation of PI3P, NOX is indispensable for producing ROS, and the V-ATPase facilitates the recruitment of ATG16L1 to the phagosome. However, how exactly these events are coordinated, the potential cross-talk, and their regulation is still obscure^287–289^. In addition, recent studies suggest that LAP plays a crucial role in antigen presentation^210,290^. Further clarity on the mechanism by which LAP participates in these processes may lie in elucidating the detailed role of V-ATPase as it is not only associated with the recruitment of key proteins but is also important for an efficient lysosomal function that effectively generates peptides for antigen presentation.

**Figure 5.**
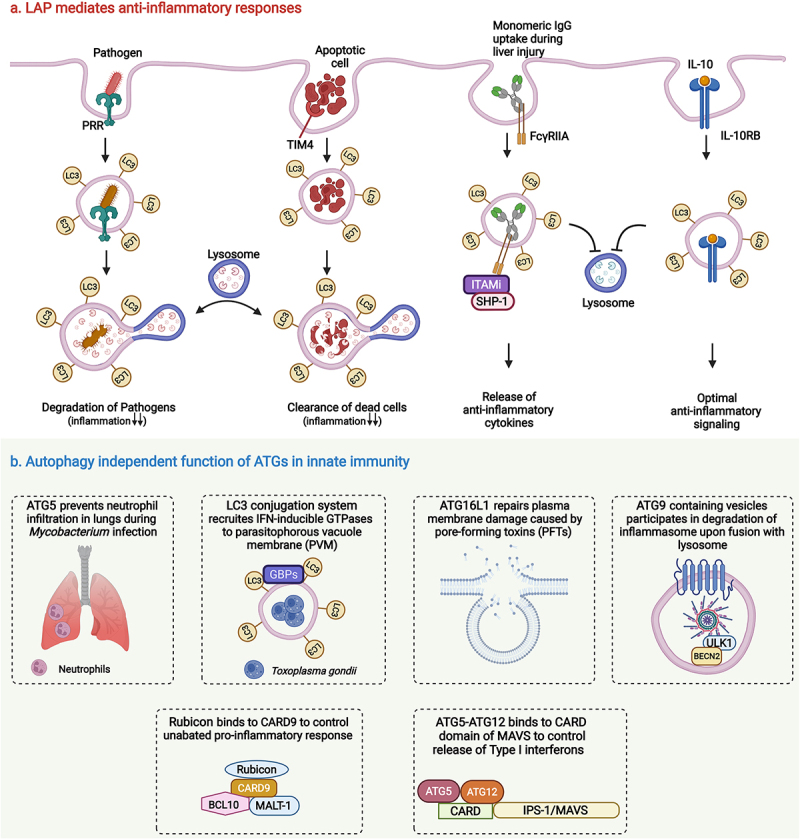
Role of unconventional autophagy and autophagy proteins in regulation of innate immune responses. (a) LC3-associated phagocytosis (LAP) mediates anti-inflammatory responses. LAP contributes to the degradation of intracellular pathogens (via PRR-mediated phagocytosis) and dead cells (via TIM4-mediated engulfment of apoptotic cells) which facilitate to dampen the inflammatory responses. As opposed to its degradative role, LAP also promotes the assembly of several anti-inflammatory signaling complexes (FcγRIIA-SHP-1-ITAMi and IL-10-1L10-RB) to control the levels of inflammation (b) Autophagy independent functions of ATGs in innate immunity. Several core autophagy proteins, independent of their autophagic function, regulate inflammatory responses. ATG5 prevents neutrophil infiltration in the lungs during *Mycobacterium* infection to control hyper inflammation and induce cell survival. LC3-conjugation system facilitates the targeting of guanylate-binding proteins (GBPs) on the *Toxoplasma* parasitophorous vacuolar membrane, thereby damaging and destroying the vacuolar niche of the parasite. ATG16L1 contributes to plasma membrane repair upon infection of pore-forming toxins (PFTs) producing bacteria, thus providing resiliency towards PFTs. ATG9 containing vesicles are involved in lysosomal mediated degradation of inflammasome to regulate inflammation, the assembly of inflammasome sensors into atg9 containing vesicles is dependent on Beclin2 and ULK1. Rubicon and ATG5-ATG12 complex control proinflammatory responses by binding to CARD9 and MAVS respectively.

LAP limits pro-inflammatory responses by degrading phagocytosed dead cells. TIM4, a phosphatidylserine receptor essential for this process, induces rapid translocation of LC3 to the phagosome-containing cell corpses (Figure 5a). This promotes phagosome acidification and effective degradation of engulfed apoptotic and necrotic cells^291^. In 2016, the same group performed *in vivo* experiments and showed that LAP-lacking animals display an increased level of inflammation, autoantibodies, kidney damage, and a systemic lupus erythematosus (SLE) like phenotype upon repeated injection of dead cells^292^. This highlights the importance of LAP in dampening inflammation. LAP is also a significant contributor to efferocytosis, a process by which dead cells are cleared. Disruption of LAP upon long-term exposure to cigarette smoke (CS) results in failed efferocytosis, thus contributing to Chronic obstructive pulmonary disease (COPD). CS exposure reduces Rubicon levels, which perturbs the LAP pathway, causing severe lung inflammation and damage^293^.

Furthermore, the LAP pathway in monocytes has been reported to reduce inflammation during cirrhosis. However, patients with acute-on-chronic liver failure (ACLF) showed negligible LAP levels. Patients affected with liver diseases show elevated serum monomeric IgG levels. Surprisingly, the introduction of intravenous monomeric IgG in ACLF patients restores LAP via activation of the FcγRIIA-SHP-1-ITAMi signaling axis to impart anti-inflammatory responses^294^ (Figure 5a). However, exactly how LAP facilitates the assembly of anti-inflammatory signaling complex upon exposure to monomeric IgG is yet to be explored.

The C-terminal WD40 domain of ATG16L1, critical for LAP but not for canonical autophagy^295^, is a key regulator of anti-inflammatory cytokine signaling. Cytokine receptors interact with ATG16L1 WDD via a WDD-binding motif, which facilitates the LC3 lipidated compartmentalization of anti-inflammatory cytokines and their receptors (such as IL-10/IL-10RB) for optimal signaling instead of degradation (Figure 5a). Interestingly, this process is independent of Rubicon^296^. Though all the above studies point towards the anti-inflammatory function of LAP in monocytes/macrophages, a small number of studies have also highlighted the pro-inflammatory effects of LAP. FcγR-mediated phagocytosis of DNA-specific IgG autoantibodies (hallmarks of autoimmunity) by plasmacytoid dendritic cells (pDCs) leads to excessive secretion of IFN-α. Henault et al. showed that LAP is essential for IFN-α secretion in response to DNA autoantibodies. LAP drives the trafficking of TLR9 (DNA sensor) containing phagosomes to a mature compartment (late endosomes), wherein the interferon signaling components interacts (TLR9, TRAF3, IRF7, etc.). This results in the pathogenic production of Type I interferons from pDCs, suggesting a role for LAP in autoimmunity^297^. *Histoplasma capsulatum*, a fungal pathogen that causes pulmonary mycosis in immunocompromised individuals, is recognized by the PRR Dectin-1. Dectin-1 activates the downstream effector, Syk, to generate ROS through NADPH oxidase in macrophages. Besides ROS, the NLRX1-TUFM-ATG5-ATG12 axis also promotes LAP induction. Eventually, LAP activates the MAPK-AP-1 pathway and cytokine production in a Rubicon-independent manner, contributing to anti-fungal immunity^298^. Although, the mechanistic details are lacking as to how LAP activates the MAPK-AP-1 pathway.

## 5. Unconventional functions of Atg proteins

Several autophagy proteins have been reported to play role in inflammation regulation without the involvement of the autophagy pathway. Herein, we highlight their unconventional roles in the hierarchical order as they participate in different stages in the autophagy pathway (Figure 5b).

*Mycobacterium* infection in mice depleted of Atg5 in myeloid cells results in aberrant infiltration of neutrophils in the lung, independent of its role in autophagy. This leads to hyper inflammation, bacterial proliferation, and reduced survival^299^. However, the molecular mechanism is yet to be discerned for this phenotype. Another study by Choi et al., showed the role of LC3 conjugation machinery in the recruitment of IFN-γ effectors on LC3 coated parasite containing vacuoles independent of their role in degradation. *Toxoplasma gondii*, a protozoan parasite, survives inside a single membrane parasitophorous vacuole membrane (PVM) by avoiding fusion with lysosomes. Upon IFN-γ stimulation, PVMs of *T. gondii* conjugate with LC3 by ATG12-ATG5-ATG16L1 complex independently of ULK1/PI3K complex. LC3 conjugation to PVMs results in the recruitment of IFN-inducible GTPases. IFN-inducible GTPases damage PVMs and subsequently restrict parasite replication^300^. Pathogens such as *Listeria* and *Streptococcus* often use pore-forming toxins (PFTs) to promote dissemination by damaging plasma membrane permeability and epithelial barriers^301^. Tan et al. showed that autophagy protein ATG16L1 and its interactors ATG5 and ATG12 are essential for providing resistance to PFTs by repairing plasma membrane independent of their autophagy function. ATG16L1 triggers lysosomal exocytosis and bleb formation to repair PM dependent on cholesterol trafficking towards PM^302^.

Rubicon acts as a specific feedback inhibitor of CARD9, an adaptor protein that plays a vital role in providing innate immunity against infection. CARD9 forms a downstream signaling complex with BCL10-MALT-1 upon recognition of PAMPs by PRR and induces the production of proinflammatory cytokines. Rubicon directly interacts with CARD9 to control its unabated activation and eventually inflammation upon specific stimulation (β-1,3-glucan or SeV infection). The Rubicon-CARD9 interaction is functionally and genetically separate from Rubicon’s role in canonical and non-canonical autophagy^303^. Another autophagy protein, Atg9a, plays a distinct role in regulating the translocation of STING (cytosolic DNA sensor) and assembly of STING and TBK1 upon dsDNA stimulation. This prevents the uncontrolled release of cytokines^304^. BECLIN2 (BECN2) negatively regulates inflammasome activation in an Atg9a and ULK1, not ATG16L1, LC3, and BECLIN1 dependent manner. ULK1 serves as an assembling factor of inflammasome sensors and BECN2 in Atg9a containing vesicles. Further, several SNAREs (SEC22A, STX5, and STX6) drive membrane fusion for inflammasome degradation to control inflammation^305^.

Autophagosomes serve as a replicative platform for several RNA viruses^306,307^. RIG-I or MDA5 sense the cytosolic viral RNA, which exposes their two amino-terminal caspase activation and recruitment domains (CARDs). Upon viral RNA detection, RIG or MDA5 interacts with the CARD domain of mitochondrial antiviral signaling protein (MAVS/ IPS-1). This interaction activates the downstream signaling cascade to activate the transcription of type I interferon genes, an essential factor contributing to antiviral host response. Intriguingly, Atg5-Atg12 conjugate associates with the CARD domain of IPS-1 upon RNA virus infection to suppress type I interferon response. RNA viruses have evolved to leverage this interaction, thus sabotaging the host’s innate antiviral immunity for their survival. The authors have also highlighted the importance of this atypical role of Atg5-Atg12 to maintain immune homeostasis under physiological conditions^308^. Nevertheless, it remains to be identified which domain or amino acid of Atg5 is required for interaction with IPS-1.

These studies highlight the non-canonical role of autophagy and autophagy proteins in regulating inflammatory response to maintain immune homeostasis. However, a clearer distinction and better mechanistic understanding will be required before unconventional autophagy pathways can be targeted for therapeutic intervention in inflammatory diseases.

## 6. Autophagy and innate immune crosstalk: disease perspective

**Figure 6.**
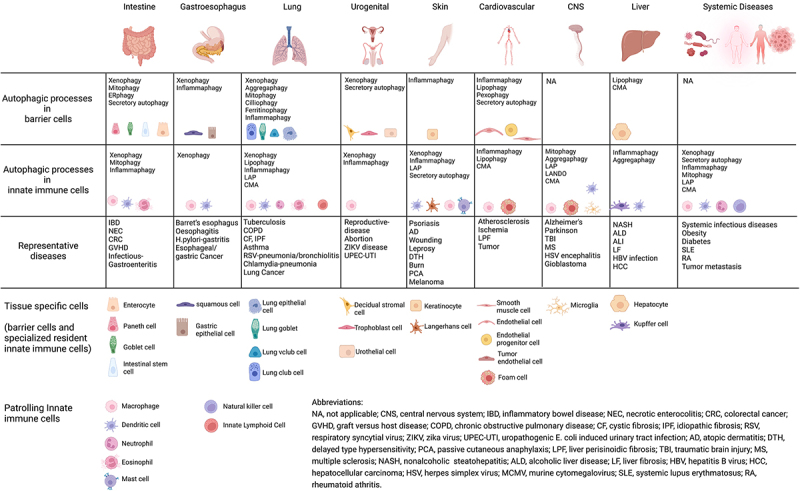
Autophagic processes regulate innate immunity in diseases. Innate immunity in organs is mediated by autophagic processes occurring in both tissue-specific barrier cells and innate immune cells. Alterations in autophagic processes are linked to a variety of organ-specific or systemic diseases. These autophagic processes include macroautophagy, LC3-associated phagocytosis (LAP), LC3-associated endocytosis (LANDO), secretory autophagy, and chaperone-medidated autophagy (CMA). Macroautophagy processes can be further classified as selective autophagy processes such as xenophagy (targeting intracellular microbes), aggregaphagy (targeting protein aggregates), mitophagy (targeting mitochondria), ERphagy (targeting endoplasmic reticulum), pexophagy (targeting peroxisome), lipophagy (targeting lipids), cilliophagy (targeting cilia), and ferritinophagy (targeting ferritin). Autophagic processes associated with regulating innate immunity, either within barrier cells or specialized innate immune cell subsets, and the representative diseases reviewed in this article are grouped by the organs listed.

As the first line of defense, our innate immune system consists of barrier tissue and its resident immune cells as well as patrolling leukocytes that can be mobilized swiftly to neutralize the threat. Barrier systems can act as a physical wall consisting of cells connected through tight junctions that insulate intruders, which are reinforced by chemical weapons and armor, such as secreted antimicrobials and mucus. Both the structural and immune cells present in barrier tissue activate innate immune responses following stimulation of pattern recognition receptors (PRRs). Activation of signaling cascades downstream of PRRs leads to a series of effector mechanisms such as secretion of soluble factors (cytokines, chemokines, and microbicidal agents), adjustment of barrier permeability, regeneration after injury, intracellular restriction/processing of hazards, triggering cell death and initiation of adaptive immunity. Autophagy, as a cardinal mediator of cellular homeostasis, is fundamental to both the process of PRR signaling and these downstream effector mechanisms. As such, a broad spectrum of diseases has been linked to dysregulated autophagy in innate immunity. In this section, we highlight the autophagy-innate immunity crosstalk from a disease perspective (Figure 6).

### A. Autophagy regulation of barrier sites in diseases

#### Gastrointestinal tract

The gastrointestinal (GI) tract represents an enormous mucosal surface area that requires protection from infectious and non-infectious foreign material ingested along with food and water. The GI tract is also persistently colonized by a massive community of resident microbes, the microbiota. A role for autophagy in supporting a balanced immune reaction at this interface is supported by the association between variants in autophagy genes (*NOD2, ATG16L1, IRGM, ULK1*, and *LRRK2*) and inflammatory bowel disease (IBD)^309^, a debilitating disease of the GI tract that involves abnormal reactions against microbes. Additionally, autophagy activity is regulated by innate immune signaling at the intestinal barrier. For example, both commensal and invasive bacteria, or bacterial products, can activate PRRs such as NOD2 to induce autophagy or autophagy-related processes by recruiting the core autophagy machinery^41–44–310–315^. LC3 conjugation occurs constitutively in the epithelium due to the presence of the microbiota and can be further enhanced to control *Salmonella enterica* Serovar Typhimurium (STm) infection^311,312^. Factors associated with damage such as HMGB1, can also induce autophagy^316,317^. Autophagy genes can be transcriptionally regulated, as demonstrated by the upregulation of ATG16L1 by the vitamin D receptor to maintain Paneth cells, an epithelial cell that secretes antimicrobial molecules to protect adjacent intestinal stem cells (ISCs) in the small intestinal crypts^318,319^.

ATG16L1 and autophagy proteins are necessary for the viability and function of Paneth cells. Patients with a major type of IBD called Crohn’s disease who are homozygous for the T300A variant of *ATG16L1* display morphologically aberrant Paneth cells, which is reproduced in mice harboring mutations in various autophagy genes^320–329^. ATG16L1 T300A protein is prone to caspase-3 mediated processing and degradation, suggesting that individuals harboring this risk variant may have reduced capacity for autophagy or an autophagy-related pathway under conditions in which caspases are activate^330,331^. Paneth cell function is supported by the role of autophagy in countering organelle stress and mediating secretion of the antimicrobial protein lysozyme when the ER-Golgi secretory pathway is disrupted by infections^321,332^ or mutations in the unfolded protein response (UPR) genes^333–335^. Autophagy also prevents cell death of intestinal epithelial cells, and Paneth cells are particularly vulnerable to inflammatory forms of cell death such as necroptosis upon inhibition of autophagy proteins or mitophagy^314,336–339^. Thus, deleting autophagy specifically in Paneth cells leads to inflammatory events resembling IBD^325^.

The function of autophagy proteins in other epithelial cells also contribute to supporting the integrity of the intestinal barrier. In a manner resembling the above role in Paneth cells, autophagy controls ISC homeostasis by limiting toxic ROS production, which is critical for epithelial regeneration after irradiation^340^. In mucus-secreting goblet cells, the autophagy machinery is mobilized downstream of another NOD-like protein, NLRP6, to mediate the exocytosis of mucin-containing granules, a process that is dependent on FOXO1 and NADPH oxidase activity^99,341–345^. More direct functions of autophagy proteins in supporting the physical barrier include breaking down the tight junction protein Claudin-2^346^, regulating proliferation^347^, and maintaining plasma membrane integrity^348^. The latter involves activating the ATG16L1 complex in response to damage by *Listeria monocytogenes* through a mechanism independent of an autophagosome. Therefore, autophagy proteins have a major role in supporting the intestinal barrier through autophagy-dependent and -independent processes.

The above processes involved in supporting the viability and function of intestinal epithelial cells are complemented by the role of autophagy in tuning the innate immune response to gut microbes, especially the production of inflammatory mediators such as interferons and interleukins. ATG16L1 deficiency in the epithelium leads to augmented type I interferon responses that enhance resistance to the model enteric pathogen *Citrobacter rodentium*^349,350^ but increases susceptibility to TNF induced cell death^339,351^. Similarly, knockout of *Epg5*, which is involved in autophagosome maturation and mutated in a combined immunodeficiency called VICI syndrome^352,353^, increases resistance to enteric virus infection via elevated type III interferon^354^. Selective autophagy receptors Optineurin and NDP52 affect IBD by regulating proinflammatory cytokines release and TLR-NF-κB activation, respectively^355–357^. In rodent models of necrotizing enterocolitis (NEC), an intestinal inflammatory disorder associated with preterm birth, excessive autophagy downstream of innate immune responses contributes to disease, suggesting a pathologic role for the pathway in this context^358–360^. Whether autophagy prevents or promotes intestinal malignancies depends on the stage and context of tumorigenesis^361^. For example, autophagy deficiency suppresses tumor growth in the setting of APC-haploinsuffiency^362^. However, increased epithelial autophagy activity by colibactin-producing *Escherichia coli* (CoPEC) is protective in a similar APC^min/+^ colorectal cancer (CRC) model^363^. Also, mitophagy in IECs promotes iron accumulation in lysosomes that trigger lysosomal membrane damage and leakage of proteases into the cytosol, which in turn enhances MHC-I antigen presentation of processed peptides that boost the antitumor CD8 T cell response^364^.

In addition to events affecting the small intestine and colon, upper GI tract diseases in the esophagus and stomach are associated with autophagy dysregulation. Barret’s esophagus, an inflammatory condition of the esophagus caused by chronic gastric acid reflux, is associated with altered autophagy activity^365,366^. Autophagy is important for epithelial cell survival in eosinophilic oesophagitis^367^, but high autophagic activity is associated with a poor prognosis of esophageal squamous cell carcinoma^368,369^. In the context of gastric *Helicobacter pylori* (*H. pylori*) infection, autophagy-induced drugs help control infection^370^ and the autophagy-compromising ATG16L1 T300A variant exacerbates inflammation^371^. However, increased autophagy might contribute to *H. pylori*-associated gastric cancer via promoting cancer cell stemness^372,373^. These examples highlight how the homeostatic function of autophagy is generally protective but occasionally contributes to disease pathogenesis, especially when persistent inflammation is involved.

#### Respiratory tract

The respiratory tract represents another major mucosal barrier site where important functions of autophagy have been documented. Autophagy processes, including ciliophagy (targeting airway epithelial cilia)^374,375^, ferritinophagy (targeting ferritin)^376^, mitophagy-mediated necroptosis^377^ and autophagy induced apoptosis^378,379^, have been proposed to contribute to chronic obstructive pulmonary disease (COPD). Particulate matter triggered lung injury also requires autophagy for disease onset^380^. However, during cystic fibrosis (CF), a disease associated with opportunistic infections and mucosal barrier abnormalities due to mutations in cystic fibrosis transmembrane conductance regulator (*CFTR*), defective autophagy is associated with more severe disease^381,382^. Aggregaphagy (autophagy targeting protein aggregates), xenophagy (autophagy targeting intracellular microorganisms), and autophagic restriction of inflammation (inflammophagy) may also safeguard against CF progression^383,384^. Similar protective functions of autophagy are found in idiopathic pulmonary fibrosis (IPF)^385,386^, such as PINK1/PARK2-mediated mitophagy in alveolar epithelial cells type 2^387–389^. In asthma, another major pulmonary disease, impaired autophagy in epithelial cells contributes to eosinophilic inflammation in obese asthmatic mice^390^. Alveolar epithelial-selective autophagic degradation of the E3 ligase TRIM37 leads to stable levels of its target TRAF6, which mediates NF-κB signaling and chemokine production to recruit neutrophils that promote lung cancer metastasis in response to particulates^391^. Similar to the intestine, lung epithelial stem-like vClub cells require autophagy for tissue repair under disease conditions such as allergic inflammation^392^. Secretion of mucus by lung goblet cells is also mediated by autophagy, and excess autophagy activity predisposes mice to type 2 airway diseases^393,394^. The secretion of defensive proteins in Club cells is also mediated by autophagy and defective autophagy is associated with COPD^395^. One interpretation of these collective findings is that autophagy can be either pathologically augmented or reduced during lung disease, and in either case, innate immune mechanisms intrinsic to the epithelium can become dysregulated, such as inappropriate production of soluble inflammatory mediators and mucus.

#### Urogenital tract

In the urogenital system, autophagy controls inflammation to prevent complications during pregnancy, such as by dampening the NLRP3 inflammasome^396,397^. Autophagy in decidual stromal cells is also important to maintain local natural killer cells that are important for preventing spontaneous abortion^398^. Autophagy in the epithelial barrier is necessary for defense against urogenital pathogens. Likely due to the role of the autophagy machinery in rearranging membranes subverted by viruses for replication, autophagy deficiency in mouse trophoblasts renders the host resistant to Zika virus^399^. Analogously, deficiency of ATG16L1 or ATG7 in the urothelium lining of the urinary tract, or carriage of the ATG16L1 T300A allele, interferes with vesicle trafficking events subverted by uropathogenic E. coli (UPEC) for persistence^400^.

#### Skin

Autophagy deficiency leads to the compromised organization of skin barriers because autophagy governs the development and proliferation of epithelial cells, and also mediates the proper inflammatory status in response to purturbations^401–404^. For example, keratinocyte autophagy is required for suppressing inflammation and facilitating wound healing, and resisting particulate matter-induced dermal fibroblast injury^405–408^ and psoriasis^409–411^. In atopic dermatitis (AD) patients, increased autophagy activity was observed in skin epithelial cells, potentially as a compensatory mechanism to mitigate inflammation^412^.

#### Endovascular barrier

The endovascular system serves as both a barrier and conduit for the trafficking of immune cells. Autophagy dysfunction in endothelial cells has been shown to instigate a variety of diseases^413^. For example, the endothelium requires autophagy to maintain integrity via controlling ROS levels in the cell^414^, promoting angiogenesis^415^, and secreting blood clotting factors^416^. Notably, endothelial cell autophagy declines with aging, and decreases in specialized functions of autophagy such as lipophagy are associated with degenerative arterial disease, like atherosclerosis^417–420^. Targeting of peroxisomes by autophagy (pexophagy) in endothelial cells is important to prevent LPS-induced organ injury^421^. During *Staphylococcus aureus* infection, endothelial-intrinsic ATG16L1 is important to tolerate damage caused by a bacterial pore-forming toxin called α-toxin^422^. Mechanistically, the secretory autophagy pathway mediates the exocytosis of decoy exosomes termed defensosomes that are decorated by the toxin receptor ADAM10, which binds and inhibits α-toxin^423^. In the blood-brain barrier (BBB), endothelial autophagy has been shown either to prevent ischemia-induced barrier damage by reducing apoptosis^424,425^ or to exacerbate brain ischemia BBB damage in diabetic mice by degrading the junction protein claudin-5^426, 427^ suggesting a context-specific role of endothelial autophagy at this site. Deletion of autophagy genes in liver sinusoidal endothelial cells renders these cells susceptible to increased inflammatory responses in the setting of non-alcoholic steatohepatitis (NASH)^428^.

### B. Autophagy regulation of innate immune cells in diseases

In addition to directly functioning within barrier cells, autophagy in innate immune cells such as macrophages, dendritic cells (DCs), granulocytes and innate lymphoid cells (ILCs) affects both tissue-specific and systemic innate immune responses.

#### Gastrointestinal tract

ATG16L1 deficiency in hematopoietic or myeloid cells renders mice more susceptible to experimental colitis due to hyperactivation of pan inflammatory responses including the inflammasome and IL1β/IL18 processing^429–432^. Excess IL-1 activity also mediates colitis in chronic granulomatous disease patients and the mouse model, which is caused by loss of function mutations in the NADPH oxidase and impaired recruitment of LC3 to the phagosome^433^. Macrophages in anti-TNF treated IBD patients display increased autophagy activity concomitant with “M2-like” anti-inflammatory features, but this effect is impaired by the ATG16L1 T300A variant^434^. However, this role of autophagy in polarizing macrophages towards an anti-inflammatory state may support CRC by suppressing antitumor immunity^435^. Nevertheless, therapeutically increasing autophagy including mitophagy can enhance resistance to CRC in the murine model by dampening the inflammasome^436,437^. DCs and macrophages without SQSTM1 or TAX1BP1 selective autophagy have amplified TLR-TRIF-IFNβ signaling, which is associated with unresponsiveness to anti-TNF treatment in IBD patients^22,23^. In myeloid cells, the absence of ATG5 will shunt selective autophagy receptor NBR1 to target IL-12 to late endosomes for secretion, thus instigating inflammation in a murine colitis model^438^. In addition to controlling inflammation, autophagy proteins in myeloid cells affect intestinal diseases in many other ways. For example, macrophages use the autophagy gene NRBF2 to scavenge apoptotic cells by enhancing phagosome maturation during intestinal inflammation^439^. Eosinophil autophagy is required for eosinopoiesis but impedes effector function upon *C. rodentium* challenge^44^°. During STm infection, LAP induced by flagellin-TLR5 activation in zebra fish macrophages delivers antimicrobial activity^440,441,442^. Also, the V-ATPase subunits on STm containing phagosomes mediate xenophagy by promoting phagosome-lysosome fusion, and loss of ATP6V0D2 in macrophages leads to impaired bacterial control in vivo after gastrointestinal infection^432^. This finding is corroborated by another study that demonstrates that V-ATPase can target ATG16L1 to STm containing vesicles for xenophagy^443^. Autophagy in intestinal DCs is important for proper interactions with T cells, as impaired autophagy and the ATG16L1 T300A variant prolong their interactions through the immunological synapse leading to hyperactivation of T cells^444^. DC autophagy deficiency leads to enhanced levels of T cell costimulation and exacerbated intestinal damage in a mouse model of graft-versus-host disease (GVHD)^234^. In the upper GI tract, *H. pylori* infection can induce xenophagy in macrophages and DCs which is impaired in PBMCs from individuals with the ATG16L1 T300A variant ^445–449^.

#### Urogenital tract

Although mechanistically distinct from how autophagy deficiency in the urothelium is protective, myeloid-specific deletion of ATG16L1 strongly enhances resistance to urinary tract infection by UPEC. This enhanced immunity is dependent on increased NLRP3/IL-1β signaling^450^. DC depletion of ATG5 increased disease pathology and morbidity in mice during vaginal HSV infection due to impaired T cell priming^224^.

#### CNS

Neuronal cell-intrinsic autophagy dysfunction has been well documented to associate with neurodegenerative diseases (including Alzheimer’s disease, AD, and Parkinson’s disease, PD). Experimental models implicate an essential role for autophagy and related processes in removing harmful extra- or intracellular protein aggregates and large organelles and neuronal cell death^451–456^. In microglia, which are myeloid cells in the CNS, LANDO is critical for β-amyloid (Aβ) receptor recycling from endosomes to the plasma membrane, which mediates removal of accumulated Aβ in the tissue^457,458^. Selective autophagy mediated by SQSTM1 in microglia removes α-synuclein aggregates released by neurons^459^. Autophagy and chaperone-mediated autophagy (CMA), which is upregulated after traumatic brain injury or spinal cord injury in neuronal cells including microglia^460,461^, both control inflammasome activation to prevent PD-like symptoms in mice^462,463^. In glioblastoma, pericytes upregulate their CMA for acquiring an immunosuppressive feature to promote tumor growth^464^. Autophagy is also important in multiple sclerosis (MS), a chronic inflammatory condition in the CNS^465,466^. Myeloid/DC autophagy (including mitophagy, lipophagy) or LAP inhibition reduces experimental autoimmune encephalomyelitis (EAE), a mouse model of MS, through multiple mechanisms^467–475^. Intriguingly, a gain-of-function variant of the mitophagy receptor NDP52 is associated with improvement in MS patients by reducing proinflammatory cytokines production^476,477^. In HSV-induced encephalitis, patients with loss of function mutations in *ATG4A* and *LC3B2* have a defect in autophagy induction, explaining increased viral replication and recurrent clinical symptoms^478^. These findings support results obtained in animal models implicating autophagy proteins in the control of herpes viruses. As further support of interactions between the virus and autophagy, the HSV encoded factors ICP34.5 and ICP0 interfere with BECLIN1 and SQSTM1/Optineurin, respectively^479,480^.

#### Lung

*Mycobacterium tuberculosis* (*Mtb*) infects macrophages, and autophagy has been shown to both limit active *Mtb* infection and the inflammation *in vitro* and *in vivo*^197,481^. *Mtb* encodes virulence factors ESAT-6 and CpsA that interfere with autophagy and LAP, respectively, to promote survival in macrophages^482^. In contrast to macrophages, ATG5 has a unique protective role in mediating the proper turnover of neutrophils independent of other autophagy proteins, which is necessary to limit immunopathology of the lung during *Mtb* infection^483^. During Chlamydia pneumoniae infection, autophagy is required to restrict inflammasome activation to restrict infection *in vivo*^484^. Autophagy in ILC2 is required for disease development in a mouse model of allergic asthma; inhibiting ATG5 in activated ILC2s increases apoptosis due to a deficit in lipophagy and a consequent metabolic shift^485^. In contrast, autophagy in DCs is protective against airway inflammation induced by the respiratory syncytial virus (RSV) by ensuring proper antiviral responses through T cell polarization^486,487^. CMA limits lung cancer progression in a mouse model through regulating macrophage activation by degrading ERK3^488^.

#### Skin

Autophagy has been shown to promote antigen presentation by Langerhans cells, a type of macrophage in the skin, to restrict *Mycobacterium leprae* infection^489^. LAP in dermal DCs is required for immunosuppression after UV exposure and contact hypersensitivity^490^. Vitamin D-induced autophagy activity in skin macrophages improves sunburn resolution by promoting wound healing macrophage differentiation^491^. Skin macrophage autophagy also protects against the treatment of mice with the TLR7 agonist imiquimod, a model of psoriasis, by inhibiting NF-κB^492^. However, ATG7 and LC3 targeting is necessary for mast cell degranulation and sustains inflammation in passive cutaneous anaphylaxis^493^. Autophagy inhibition in melanoma cells also renders improved tumor killing by NK cells via upregulating chemokine CCL5 expression, as autophagy deficiency leads to PP2A deactivation and thus promotes JNK-c-Jun-CCL5 axis activity^494^.

#### Liver

Patients with chronic HCV infection display heightened autophagy activity compared to other liver disease patients^495^. This upregulation of autophagy, including CMA, is associated with control of lipid storage in hepatocytes^496^, and resistance to IFN-I and ribavirin treatment due to downregulation of IFNAR1 and nucleoside transporters^497–501^. During acute liver injuries, autophagy is induced in Kupffer cells, the liver type of macrophages, and chemical inhibition or myeloid ablation of ATG5 increases disease with elevated inflammatory response^502–504^. The Kupffer cell autophagy is also protective in alcoholic liver disease^505^, liver fibrosis^506^, and hepatocarcinogenesis^507^ in mice via restraining ROS-induced IL-1α and IL-1β production^506^. In the context of hepatocellular cancer (HCC), hepatocyte autophagy restricts tumorigenesis by reducing SQSTM1 accumulation, while autophagy in the myeloid compartment is also antitumorigenic through downregulation of immunosuppressive molecules like PD-L1^508–511^.

#### Cardiovascular disease

In atherosclerosis, macrophage foam cells use autophagy to mediate intracellular lysosomal cholesterol hydrolysis and efflux^512^ thereby reducing inflammation and plaque formation/necrosis^513,514^. These observations are corroborated by the finding that ectopic overexpression of ATG14 in macrophages decreases plaque formation in atherosclerosis-prone *ApoE^–/–^* mice^515^. Autophagy deficiency in smooth muscle cells leads to increased CCL2 secretion and recruitment of macrophages to increase plaque formation in vivo^516^. CMA activity is lower in atherosclerosis plaques of patients, and CMA controls inflammasome activation to reduce atherosclerosis in *ApoE^–/–^* mice^517,518^.

#### Obesity and diabetes

Impaired autophagy in adipose tissue is associated with an inflammatory state in individuals with obesity^519,520^ and primes insulin resistance in a mouse model^521^. In line with these observations, autophagy inhibition in macrophages exacerbates metabolic disease in genetic and diet-induced models of obesity and diabetes due to increased inflammasome and ROS production^430,522,523^. Mitophagy is specifically important for staving off obesity^524,525^. However, discrepant results have questioned the necessity of macrophage autophagy in adipose tissue lipid metabolism^526,527^, perhaps reflecting differences in techniques and models.

#### Multisystem inflammatory disease

Autophagy gene expression and markers are decreased in patients with systemic lupus erythematous (SLE)^528,529^, and the *ATG5* genetic locus is associated with disease susceptibility^530^. These findings could reflect a protective or adverse function of autophagy proteins. SQSTM1 activity in macrophages mediates cell death downstream of TLR7 that coincides with autophagy induction in a mouse model of SLE^531^, and LAP in DCs is necessary for TLR9 activation by DNA-immune complexes that underly interferon production, an autoimmune disease hallmark^297^. The same mechanisms by which autophagy dysfunction can contribute to tissue-specific innate immune hyperactivity could potentially explain multi-organ manifestations. Uveitis, a form of inflammation in the eye, occurs more commonly in IBD patients, and inhibiting autophagy in macrophages causes disease in mice through inflammasome activation and IL1β^533^. The role of autophagy in antigen-presenting cells could contribute to autoimmune diseases, such as the presentation of the citrullinated peptides underlying rheumatoid athritis^534^. Although the molecular mechanism is unclear, drugs that upregulate autophagy mitigate lethal lung damage in mouse models of sepsis independently of microbial burden^535^, highlighting the role of autophagy in tissue resilience to immune-mediated damage.

#### Tumors in non-barrier sites

Dendritic cell-specific deletion of ATG5 impairs priming of antitumor CD4 and CD8 T cells, which is associated with increased scavenger receptor CD36 and reduced MHCII-tumor antigen presentation^536^. In an ovarian cancer metastasis model, mitophagy mediates adaptation to oxidative stress in Tim4+ tumor-associated macrophages (TAMs) that interfere with anti-tumor immunity^537^. LAP supports the tolerogenic nature of TAMs by facilitating the non-inflammatory uptake of apoptotic tumor carcasses, and thus, ablation of LAP in the myeloid compartment leads to increased IFN-I and antitumor immunity^538^.

## 7. Autophagy as a pharmacological target

Several studies have highlighted the effectiveness of autophagy regulation via small molecules^539–541^. This has opened up considerable interest in the therapeutic potential of pharmacologically active molecules that work through autophagy to have beneficial effects on inflammation. Some of such studies that have used autophagy modulating small molecules in various diseases associated with inflammation have been summarized in Table 1. Although there are not many bonafide autophagy modulators known, several of the molecules, whose mechanism of action and/or targets are known, work indirectly to control autophagy flux (Table 1). Studies have also highlighted the effectiveness of combining such autophagy modulating small molecules with anti-inflammatory drugs^542,543^.

**Table 1.** Pharmacological Activators of Autophagy.

Diseases	Autophagy modulator	Mechanism	References
Pathogenic infections	Rapamycin	mTOR inhibition	544, 545
	Torin1	mTOR inhibition	546
	AICAR	AMPK activation	547
	Gefitinib	EGFR inhibition and increase lysosomal biogenesis genes	548, 549
	AR-12	Akt kinase inhibition	550
	Tat–beclin 1	GAPR-1 inhibition	551
	Nt-Arg	Induces autophagy via p62	552
	Tamoxifen	Estrogen receptor inhibition	553
	Flubendazole	Inhibits mTOR and releases Beclin1 from Bcl2-Beclin1 complex	554
	Acacetin	Promotes TFEB nuclear translocation	555
	Trehalose	Activates MCOLN1	556, 557
	Vitamin D	Activates MCOLN3	558–56°
	Carbamazepine	Depletes cellular myo-inositol, IP3 levels and activates AMPK	561
Inflammatory bowel disease (IBD)	Rapamycin/Sirolimus	mTOR inhibition	562–565
	AICAR	AMPK activation	566
	Celastrol	Suppresses PI3K/Akt/mTOR signaling	567
	Docosahexaenoic Acid	Inhibition of mTOR signaling pathway	568
	AMA0825	ROCK inhibition	569
	Azathioprine	Inhibits mTOR and activates PERK	570
	Vitamin D	Activates MCOLN1	571, 572
Lung inflammation	Carbamazepine	Depletes cellular myo-inositol and IP3 levels, activates AMPK	573, 574
	AICAR	AMPK activation	575
	Tubastatin A	Histone deacetylase 6 inhibition	576
	Mdivi-1	Mitophagy inhibition	577
	Cysteamine	Transglutaminase 2 (TGM2) inhibitor, restores Beclin1 levels	578, 579
	Epigallocatechin-3-Gallate (EGCG)	Elevates ATG12 expression	579, 580
Heart inflammation	Rapamycin	mTOR inhibition	581–584
	Metformin	AMPK activation and mTOR inhibition	585–587
	Verapamil	Depletes ATP levels	588
Liver inflammation	Verapamil	Induces autophagic flux	589
	Micheliolide	Activates PPAR-γ and p-AMPK and inhibits p-mTOR	590, 591
	Spermine	Increases ATG5 expression	591
	Epigallocatechin-3-Gallate (EGCG)	Increase phosphorylation of AMPK	592
Systemic lupus erythematosus (SLE)	Rapamycin	mTOR inhibition	593
	Hydroxychloroquine	Inhibit lysosomal activity	594
Rheumatoid arthritis (RA)	Arsenic Trioxide	Enhances autophagic flux	595
	Metformin	AMPK activation and mTOR inhibition	596
	Artesunate	Suppresses PI3K/Akt/mTOR signaling	597
	Triptolide	Inhibits autophagy	598
	Tomorou	Mechanism unclear	599
	Oridonin	Inhibits autophagy	600
	TIPTP	p22phox (subunit of NOX) inhibition	601
	Hydroxychloroquine	Inhibits lysosomal activity	543
Multiple sclerosis (MS)	Rapamycin	mTOR inhibition	602, 542
	1,25-dihydroxyvitamin D3	Elevates Beclin1 expression	603
	Hydroxychloroquine	Inhibits lysosomal activity	604
	Clioquinol	Induce autophagy	605

## Conclusions

Since the hallmark discovery of autophagy as a homeostatic mechanism induced by starvation in yeast by Yoshinori Ohsumi and co-workers, we now realize that autophagy has multiple functions in eukaryotic cells. One important and likely evolutionary conserved one is the contribution to immune defense, discussed here. The obvious possibility of autophagy to clear intercellular microbes has meanwhile been well documented and underlying molecular mechanisms have been worked out in several cases. This contributes to innate immune defense, particularly towards bacterial infection in barrier epithelia. Probably more surprisingly, autophagy also contributes to controlling the magnitude of innate immune responses and to resolve inflammation by targeting cellular components of the inflammatory cascades, and to adaptive immune responses by influencing MHC antigen presentation. The molecular details of these rather newly discovered mechanisms are beginning to emerge, and recent data suggests that several inflammatory disorders can be explained, at least in part, by dysfunctional autophagy processes. The host protective role of inflammation on the other hand is undermined by pathogens that evolved mechanisms to subvert autophagic processes.

With a deeper understanding of the molecular mechanisms and thus identification of druggable targets, we will have the opportunity of developing novel treatment options for both inflammatory and infectious diseases.^52^,^532^,^559^,^560^
